# Glacial meltwater is associated with gene-specific diversification of metal resistance genes in high Arctic soil microbiomes

**DOI:** 10.3389/fmicb.2026.1903619

**Published:** 2026-07-27

**Authors:** Franck J. Ouedraogo, Alexandre J. Poulain, Stéphane Aris-Brosou

**Affiliations:** 1 Department of Biology, University of Ottawa, Ottawa, ON, Canada; 2 Department of Mathematics and Statistics, University of Ottawa, Ottawa, ON, Canada

**Keywords:** Arctic freshwater microbiome, gene-pool diversification, glacial meltwater, metagenomics, metal resistance genes (MRGs)

## Abstract

Climate warming accelerates glacial meltwater delivery to Arctic lakes, mobilizing metals from thawing catchments and reshaping the selective landscape experienced by resident microbes. Whether these gradients leave detectable signatures of diversification in environmental resistance genes remains unclear. We investigated four metal resistance genes (*merA*, *arsC*, *cadA*, and *chrR*) in metagenomic datasets from Lake Hazen (Nunavut, Canada), the largest High Arctic freshwater lake, sampled across a natural hydrological gradient of Control, Low-runoff, and High-runoff regimes. Using a space-for-time substitution design, we combined population-genetic and codon-based approaches to quantify diversity and candidate selection signals, including nucleotide diversity, Tajima’s *D*, non-synonymous-to-synonymous diversity ratios, McDonald-Kreitman tests with outgroup-sensitivity analysis, site-level episodic selection (MEME with false-discovery-rate correction), and gene-wide tests (BUSTED and BUSTED-E) and complemented these with ortholog clustering, within-clade re-analysis, taxonomic profiling, rarefaction, and phylogenetic beta-diversity. Marked heterogeneity emerged among genes: *merA* showed increasing diversity and patterns consistent with diversification along the runoff gradient, and these signals were preserved within the largest orthologous cluster (90% of haplotypes), supporting an interpretation of within-orthogroup diversification; *cadA* displayed the strongest McDonald-Kreitman signal under low and high runoff, but its gene-wide BUSTED-E signal collapsed within a single ortholog cluster, suggesting that part of the apparent diversifying signal at the gene-family level reflects inter-subfamily heterogeneity; *chrR* exhibited the strongest regime structure but its largest orthologous cluster was dominated by Control sequences and 93% of High-regime haplotypes were affiliated with a single bacterial order (Hyphomicrobiales), indicating that the regime contrast for this gene reflects compositional turnover rather than within-lineage evolution; *arsC* remained largely consistent with neutral or purifying evolution across regimes. Because these inferences derive from metagenomic gene pools sampled across only three hydrological regimes and aggregate variants across taxa, we interpret them as exploratory, hypothesis-generating patterns rather than as demonstrations of population-level adaptation. Our findings highlight environmental resistance genes as candidate indicators of changing biogeochemical conditions in rapidly warming polar ecosystems, while underscoring the importance of orthology and community-composition controls when inferring selection from metagenomic data.

## 1 Introduction

The Arctic is warming nearly four times faster than the global average, destabilizing cryospheric reservoirs that have sequestered metals for millennia (Rantanen et al., 2022; Box et al., 2019). Permafrost thaw and glacial retreat are releasing mercury (Hg), chromium (Cr), arsenic (As), zinc (Zn), and other metals into downstream freshwater systems (Schuster et al., 2018; Hawkings et al., 2014), where they encounter microbial communities adapted to historically pristine conditions. The biological consequences of this rapid mobilization remain poorly understood, particularly at the level of microbial gene-pool diversification. Metal resistance genes (MRGs) provide a tractable system for addressing this question because their phenotype maps directly onto a measurable environmental stressor, their molecular machinery is well-characterized, and they are often encoded on mobile genetic elements that allow rapid spread between host lineages (Bruins et al., 2000; Nies, 2003).

Four MRGs are particularly relevant to Arctic biogeochemistry. The *merA* gene encodes mercuric reductase, which converts Hg(II) to volatile Hg(0) (Barkay et al., 2003); *arsC* encodes arsenate reductase, which reduces As(V) to As(III) for subsequent extrusion (Mukhopadhyay et al., 2002); *cadA* encodes a P-type ATPase that exports Zn(II), Cd(II), and Co(II) (Argüello et al., 2007); and *chrR* encodes a chromate reductase that detoxifies Cr(VI) to less mobile Cr(III) forms (Ackerley et al., 2004). These genes encode mechanistically distinct biochemical functions and differ in their genomic mobility, raising the possibility that they will respond differently to environmental metal gradients. We focused on these four genes, and their target metals (Hg, As, Zn, and Cr; *cadA* additionally exports Cd and Co), because these are the elements documented to be mobilized along the Lake Hazen runoff gradient (St. Pierre et al., 2019) and because each gene encodes a single, well-characterized, mechanistically distinct resistance pathway suitable for sequence-level analysis. Metals lacking a comparable in-system mobilization signal or a single canonical resistance determinant in these datasets (Ni, Co, Cu, Pb) were not analyzed.

Lake Hazen on Ellesmere Island, Nunavut, is the largest freshwater lake by volume in the High Arctic and a sentinel site for climate-driven change (Lehnherr et al., 2018). Its watershed exhibits a natural gradient of glacial meltwater influence: the Ruggles River outflow (no glacial input), Blister Creek (low runoff), and the Abbé and Snowgoose rivers (high runoff). This gradient produces large differences in metal concentrations across regimes. Chromium increases monotonically with runoff, spanning a 60-fold range from 0.33 μg/L in the Ruggles outflow to 19.73 ± 2.24 μg/L at the Abbé and Snowgoose rivers. Zinc shows a similar monotonic pattern, ranging from below detection limits in lake surface water to 108.07 ± 12.02 μg/L in High runoff. In contrast, total mercury follows a non-monotonic pattern with maximum concentrations in the Low runoff regime (Blister Creek: 17.56 ± 14.14 ng/L) and lower but still elevated levels in High runoff (7.07 ± 1.27 ng/L vs. 0.42 ng/L in the Ruggles outflow), reflecting differences in both glacial reservoir chemistry and watershed discharge volumes (St. Pierre et al., 2019). The system therefore offers a space-for-time substitution design, in which contemporary regimes serve as proxies for the trajectory expected under continued glacial retreat.

Inferring selection from metagenomic gene-pool data poses several well-known methodological challenges that we explicitly acknowledge and address in this study. First, metagenomic gene pools aggregate sequence variants across taxa and across potentially divergent members of each gene family, so the panmictic-population assumption underlying classical population-genetic estimators is not strictly satisfied (Ellegaard and Engel, 2016; Garud and Pollard, 2020). Second, coalescent estimators of effective population size (*N*_e_) assume neutrality; when selection is present, *N*_e_ estimates are biased downward, complicating their interpretation (Charlesworth, 2009). Third, horizontal gene transfer of MRGs via plasmids and transposons violates the vertical inheritance assumption of single-tree coalescent models (Soucy et al., 2015; Smith et al., 2019). We addressed these challenges by (i) reframing all population-genetic and codon-based statistics as descriptive summaries of standing variation in environmental gene pools rather than as formal tests of adaptation; (ii) clustering sequences into orthologous subfamilies and repeating all selection analyses within the largest cluster per gene; (iii) profiling the taxonomic affiliation of MRG-containing haplotypes and reporting community composition per regime; (iv) applying multiple-testing correction across all per-codon and per-test outputs; (v) running outgroup-sensitivity analyses for the McDonald-Kreitman test; (vi) running BUSTED alongside BUSTED-E as a sanity control for alignment heterogeneity; and (vii) replacing coalescent demographic inference (relocated to the Material) with assumption-light alternatives in the main text (Faith’s phylogenetic diversity and PERMANOVA on patristic distances).

In this descriptive, hypothesis-generating framework, we explored three patterns: (H1) nucleotide diversity (*π*) of metal resistance genes may increase along the gradient of glacial meltwater input, consistent with greater metal mobilization co-occurring with the diversification of resistant variants; (H2) selective signals on MRGs may vary with environmental metal exposure, with genes responding to enriched metals showing patterns consistent diversification that scales with concentration; and (H3) independent methods with non-overlapping assumptions may provide convergent evidence of such patterns, allowing biological interpretation despite the methodological limitations of any single approach applied to metagenomic data.

## 2 Materials and methods

### 2.1 Site and metagenomic data

Sequencing data were obtained from two published Lake Hazen surveys deposited in NCBI BioProjects PRJNA556841 and PRJNA746497 (Colby et al., 2020). We restricted analyses to soil samples to focus on terrestrial microbial responses and to maintain internal comparability across the eight soil sites: CSED, CSOIL (Control regime); LR1, LR2, LRSOIL (Low runoff regime; Blister Creek catchment); and HR1, HR2, HRSOIL (High runoff regime; Abbé and Snowgoose River catchments). Reference protein sequences for *merA*, *arsC*, *cadA*, and *chrR* were retrieved from NCBI and UniProt.

### 2.2 Sequence recovery and quality control

Raw paired-end reads were quality-trimmed with Trimmomatic v0.39 (minimum quality 20, minimum length 50 bp). Putative MRG-containing reads were identified by translated BLAST against the reference protein set using DIAMOND v2.1.7 (*E*-value < 10^−5^, percent identity ≥ 60%, query coverage ≥ 50%; Buchfink et al., 2015). We further filtered candidate sequences by enforcing the presence of conserved catalytic motifs specific to each gene family [CXXC and CPx motifs for *cadA* (Argüello et al., 2007); the *merA* active-site Cys residues (Fox and Walsh, 1983); the *arsC* redox-active CX₅R motif (Mukhopadhyay et al., 2002); and the *chrR* FMN-binding motif (Ackerley et al., 2004)], removing reads with truncated or atypical motif architecture. Sequences were translated into protein, dereplicated at 100% identity, and aligned in codon mode with MAFFT v7.526 (Katoh and Standley, 2013). Reading-frame integrity was verified by translation against the canonical reference, and putative chimeric or recombinant sequences were screened for and removed before downstream analysis. After codon-aware filtering, the final soil-only datasets comprised 242, 1,349, 74, and 1,121 unique haplotypes for *merA*, *arsC*, *cadA*, and *chrR*, respectively. Haplotypes were treated equally rather than weighted by read abundance, to avoid conflating ecological dominance with sequence diversity; the consequences of this choice are discussed in the Limitations section.

### 2.3 Ortholog clustering and within-clade re-analysis

To control for the well-known heterogeneity of resistance gene families and ensure that the diversity and selection statistics reported below are not driven by inter-orthologue heterogeneity, we clustered the motif-filtered protein sequences of each gene with MMseqs2 v15 (Steinegger and Söding, 2017) at 50, 70, 90% sequence identity, and at a data-driven elbow threshold determined from the per-gene identity distribution. The elbow thresholds retained were 0.83 for *merA*, 0.79 for *arsC*, 0.70 for *cadA*, and 0.73 for *chrR*. For each gene, we identified the largest cluster (containing 90, 40, 22, and 45% of haplotypes for *merA*, *arsC*, *cadA*, and *chrR*, respectively) and re-ran all selection statistics restricted to this within-clade sample, to assess whether qualitative patterns persist when ortholog heterogeneity is controlled.

### 2.4 Phylogenetic inference and diversity statistics

Maximum-likelihood phylogenies were inferred from each codon alignment with IQ-TREE v2.3.6 under the best-fit substitution model selected by ModelFinder (BIC criterion) and 1,000 ultrafast bootstrap replicates (Minh et al., 2020). Nucleotide diversity (*π*) was calculated using the Nei pairwise method (Nei, 1987) separately for each gene-regime combination on soil-only haplotypes, with 95% confidence intervals derived from 500 bootstrap resamples. To assess whether differences in haplotype counts could drive the regime contrasts, we additionally subsampled each gene-by-regime sample to a common haplotype depth (*n* = 19, the smallest sample, *cadA* Control) with bootstrap confidence intervals from 500 replicates. Tajima’s *D* was computed using the same haplotype sets, with significance assessed against the empirical distribution under the standard neutral model (Tajima, 1989). Faith’s phylogenetic diversity (Faith, 1992) was computed as the total branch length of the gene-regime subtree using DendroPy v4.6 (Sukumaran and Holder, 2010), and inter-regime phylogenetic structure was tested by PERMANOVA on patristic distance matrices (9,999 permutations of regime labels).

### 2.5 *N*_e_-independent selection tests

To address the circularity that arises when *N*_e_ is estimated from data under selection (Charlesworth, 2009), we applied two analyses whose statistics do not depend on *N*_e_. First, the ratio of nucleotide diversity at non-synonymous to synonymous sites (*π*_N_/*π*_S_) was computed using the Nei-Gojobori method (Nei and Gojobori, 1986), with 95% confidence intervals from 50 bootstrap resamples. Second, the McDonald-Kreitman (MK) test (McDonald and Kreitman, 1991) was applied per gene and per regime using the best NCBI BLAST hit (*E*-value < 10^−20^) as outgroup (*Cupriavidus metallidurans* plasmid CP046332 for *merA*; *Escherichia coli* CP039837 for *arsC*; *Bacillus subtilis* CP053102 for *cadA*; and *E. coli* CP040107 for *chrR*). The proportion of adaptive substitutions *α* = 1 − (*P*_N_/*P*_S_)/(*D*_N_/*D*_S_) was computed following Smith and Eyre-Walker (2002), with significance assessed by a χ^2^ test with Yates correction. To assess the sensitivity of *α* to outgroup choice, we re-implemented the MK test with codon alignment by MACSE v2.07 *enrichAlignment* (Ranwez et al., 2018) and added 1–2 BLAST-derived outgroups per gene in distinct amino-acid identity bins (close 65–72%, intermediate 55–65%, distant 40–55%), yielding 33 outgroup-by-regime comparisons in total.

### 2.6 Codon-based selection inference and multiple-testing correction

Site-level signals of episodic diversification were assesses with the Mixed-Effects Model of Evolution (MEME) (Murrell et al., 2012) implemented in HyPhy v2.5.49 (Kosakovsky Pond et al., 2020), with empirical Bayes posterior probability used to identify codons with significant evidence for selection. We applied Benjamini-Hochberg false-discovery-rate (FDR) correction across MEME per-codon *p*-values for each gene and report site fractions at *q* < 0.05 (Benjamini and Hochberg, 1995). To complement MEME with a gene-wide test robust to alignment errors at high-*ω* sites, we applied BUSTED-E (Branch-site Unrestricted Statistical Test for Episodic Diversification with Error-sink; Selberg et al., 2025), which adds an error-absorbing rate class to the BUSTED model. As a sanity control, we additionally ran standard BUSTED (Murrell et al., 2015) and compared its Likelihood Ratio Test (LRT) to the BUSTED-E LRT to assess the contribution of the error-sink rate class. MEME, BUSTED, and BUSTED-E were each run twice per gene: once on the full gene-family alignment and once on the largest ortholog cluster (within-clade).

### 2.7 Taxonomic profiling of MRG-containing haplotypes

To disentangle community-composition shifts from within-lineage evolution, we assigned best-hit taxonomic affiliation to each MRG-containing haplotype by BLASTP against the NCBI non-redundant protein database (nr, March 2026 release; *E*-value < 10^−20^, top hit retained). Affiliations were aggregated to the order level using a standardized mapping based on NCBI Taxonomy to ensure cross-gene comparability, and the resulting compositions are reported per regime. Sequences for which no high-confidence assignment could be retrieved within the wall-clock allowance were retained for sequence-level analyses but excluded from the taxonomy figure; taxonomic coverage was 100% for *merA* and *cadA*, 93% for *arsC*, and 92% for *chrR* (the 76 unassigned *chrR* sequences are all from the Low regime; see Limitations).

### 2.8 Gene-pool diversity and exchange

Bayesian coalescent estimates of the population mutation parameter *θ* = 2*N*_e_*μ* under the Coalescent Constant Population model (Kingman, 1982) using BEAST2 v2.7.6 (Bouckaert et al., 2019), and inter-regime gene-flow rates under the structured-coalescent model MASCOT (Müller et al., 2018), were preformed, but only presented in the Supplementary material. Because the panmictic-population, neutrality, and vertical-inheritance assumptions of these models are only partially met by metagenomic gene pools, we interpret *θ* and migration rates as descriptive indices of gene-pool diversity and exchange rather than as organismal demographic parameters. Convergence was assessed with three independent Markov Chain Monte Carlo (MCMC) chains of 50 million iterations each (10% burn-in, Effective Sample Size (ESS) > 200 for all parameters, inter-chain coefficient of variation < 5%).

### 2.9 Statistical analysis of metal-diversity relationships

Associations between *π* and metal concentrations were summarized descriptively by Spearman rank correlations between gene-specific *π* values and the concentration of the target metal (*merA*-Hg, *arsC*-As, *cadA*-Zn, *chrR*-Cr) across the three regimes (Colby et al., 2020; St. Pierre et al., 2019). With only three regimes, the Spearman *p*-values are not interpretable as hypothesis tests, and we report them only as descriptive directional summaries. Bayesian directional probabilities *P*(*π*_X_ > *π*_Y_) were computed from the bootstrap distributions of *π* as the proportion of bootstrap replicates in which *π*_X_ exceeded *π*_Y_, providing a complementary measure of bootstrap directional credibility. All custom scripts and configuration files are available at the repository link below.

## 3 Results

### 3.1 Haplotype recovery and overall diversity patterns

**Figure 1:**
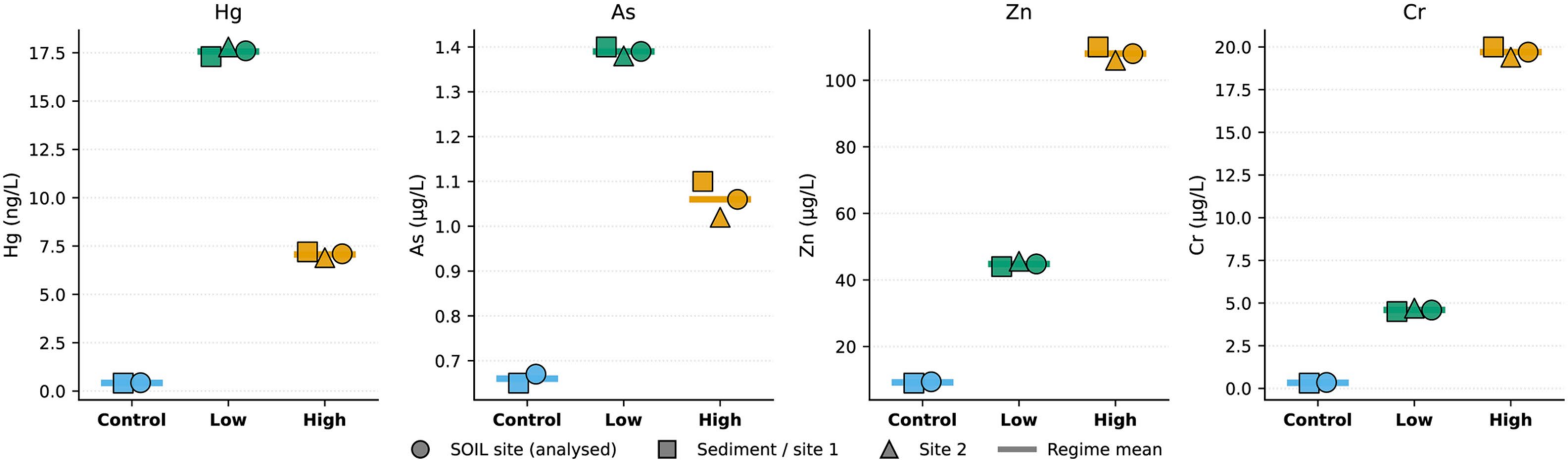
Metal concentrations across the Lake Hazen runoff gradient (St. Pierre et al., 2019). Site-level points (sediment, soil, and additional water-column sites) are shown by symbol, and the regime mean by a horizontal bar. Wong palette: control = sky blue, low = bluish green, high = orange. Concentrations are in ng/L for Hg and μg/L for As, Zn, and Cr.

**Figure 2:**
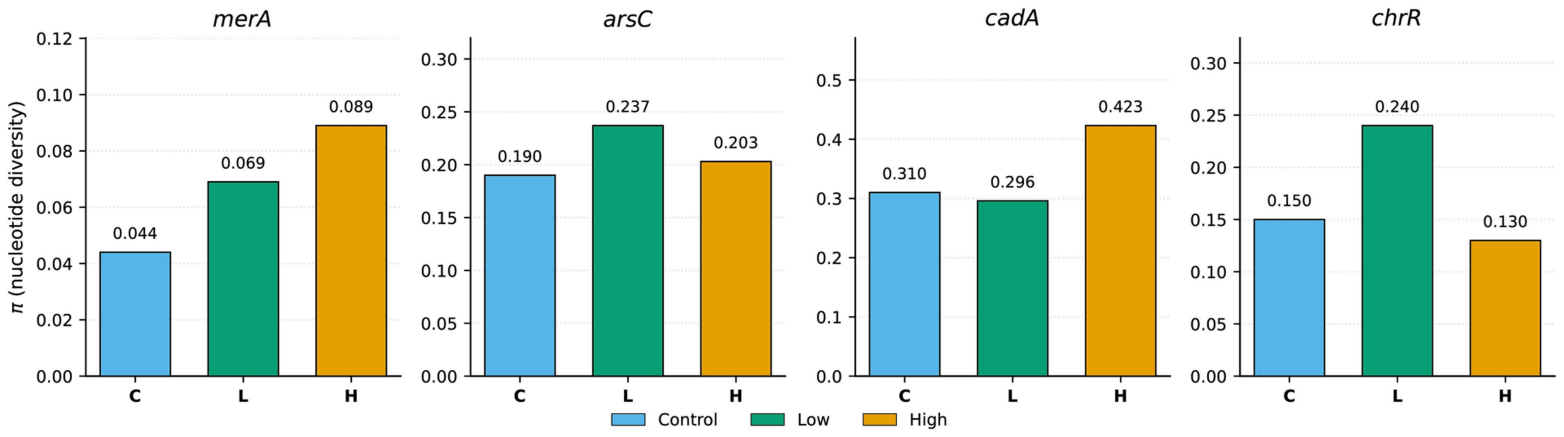
Nucleotide diversity (*π*, Nei pairwise method) per gene and regime, computed on soil-only haplotypes. Bars: regime mean; numeric labels: regime mean value. Wong palette: control = sky blue, low = bluish green, high = orange.

The complete analysis workflow is summarized in Supplementary Figure S1. After motif-based filtering and dereplication, soil samples yielded 2,786 unique metal resistance gene haplotypes: 242 for *merA*, 1,349 for *arsC*, 74 for *cadA*, and 1,121 for *chrR* (Table 1; site-level values in Supplementary Table S1). Recovery varied substantially across regimes for each gene, reflecting both natural abundance and sequencing depth. Nucleotide diversity (*π*) ranged from 0.044 (*merA*, Control) to 0.423 (*cadA*, High) and showed three distinct response patterns across the runoff gradient (Figure 1; Figure 2): *merA* increased monotonically with runoff (Control 0.044 → Low 0.069 → High 0.089; Bayesian *P* (*π*_H_ > *π*_C_) = 0.994); *cadA* reached its maximum in High (0.310 → 0.296 → 0.423; P (*π*_H_ > *π*_C_) = 0.992); *arsC* peaked in Low (0.190 → 0.237 → 0.203) consistent with an intermediate-disturbance pattern; *chrR* showed a striking non-monotonic response, with the maximum in Low (0.240) and the minimum in High (0.130), despite Cr concentrations being highest in High. Tajima’s *D* values supported these contrasts: *merA* exhibited negative values consistent with directional selection or expansion (*D* = −1.07 to −1.68, significant in Low and High; Supplementary Table S2); *arsC* and *chrR* positive values in some regimes consistent with balancing selection or population contraction (Supplementary Figure S2). To assess robustness to uneven sampling, all gene-by-regime samples were subsampled to a common haplotype depth (*n* = 19) with bootstrap confidence intervals. The qualitative direction of the regime contrasts in *π* was preserved in three of four genes (*merA*, *arsC*, *chrR*; Supplementary Table S3).

**Table 1:** Summary statistics for the four metal resistance genes across hydrological regimes at Lake Hazen.

Gene	Regime	*N*	*π* [95% CI]	Tajima’s *D*	π_N_/*π*_S_ [95% CI]	α (MK)
*merA*	Control	60	0.044 [0.036–0.054]	−1.07	0.220 [0.192–0.237]	+0.488 ***
	Low	99	0.069 [0.047–0.097]	−1.68 *	0.245 [0.210–0.277]	+0.346 **
	High	83	0.089 [0.064–0.125]	−1.63 *	0.266 [0.228–0.323]	+0.321 **
*arsC*	Control	550	0.190 [0.181–0.200]	+1.08	0.337 [0.325–0.348]	−0.380 ns
	Low	276	0.237 [0.223–0.250]	+1.90 *	0.449 [0.421–0.476]	+0.007 ns
	High	523	0.203 [0.194–0.211]	+1.35	0.452 [0.432–0.467]	−0.371 ns
*cadA*	Control	19	0.310 [0.198–0.389]	+0.24	0.866 [0.804–0.982]	+0.533 ***
	Low	29	0.296 [0.210–0.370]	+0.19	0.856 [0.757–0.903]	+0.649 ***
	High	26	0.423 [0.394–0.441]	−0.17	0.607 [0.521–0.699]	+0.538 ***
*chrR*	Control	726	0.150 [0.143–0.157]	+0.20	0.576 [0.554–0.589]	+0.198 ns
	Low	76	0.240 [0.218–0.265]	+0.79	0.454 [0.422–0.496]	+0.169 ns
	High	319	0.130 [0.118–0.143]	−0.42	0.630 [0.596–0.661]	+0.362 *

*N* = Number of unique soil haplotypes. *π* reported as mean [95% bootstrap CI]. Tajima’s *D* significance: * *p* < 0.05; *ns* not significant. *π*_N_/*π*_S_ = Nei-Gojobori ratio with 95% bootstrap CI. *α* = McDonald-Kreitman proportion of adaptive substitutions; significance from χ^2^ test with Yates correction (*** *p* < 0.001, ** *p* < 0.01, * *p* < 0.05, *ns* not significant).

### 3.2 Differential selective constraint independent of *N_e_* (π_N_/π_S_)

Nei-Gojobori *π*_N_/*π*_S_ ratios revealed gene- and regime-specific patterns of selective constraint (Supplementary Table S4). All four genes showed ratios below 1 in all regimes, indicating dominant purifying selection across the dataset: *merA* exhibited the strongest constraint (0.220, 0.245, 0.266 across Control, Low, High), with a slight monotonic relaxation along the runoff gradient; *arsC* showed an increase from Control (0.337) to metal-exposed regimes (Low: 0.449; High: 0.452), indicating relaxed purifying selection under metal stress; *cadA* showed striking regime-specific reinforcement: *π*_N_/*π*_S_ dropped from 0.866 in Control and 0.856 in Low to 0.607 in High, a 30% reduction consistent with strong purifying selection on the Zn-binding ATPase under high Zn exposure; *chrR* showed a non-monotonic pattern with a minimum in Low (0.454) and elevated values in Control (0.576) and High (0.630) (Supplementary Figure S3).

### 3.3 Non-synonymous versus synonymous divergence from the McDonald-Kreitman test

**Figure 3:**
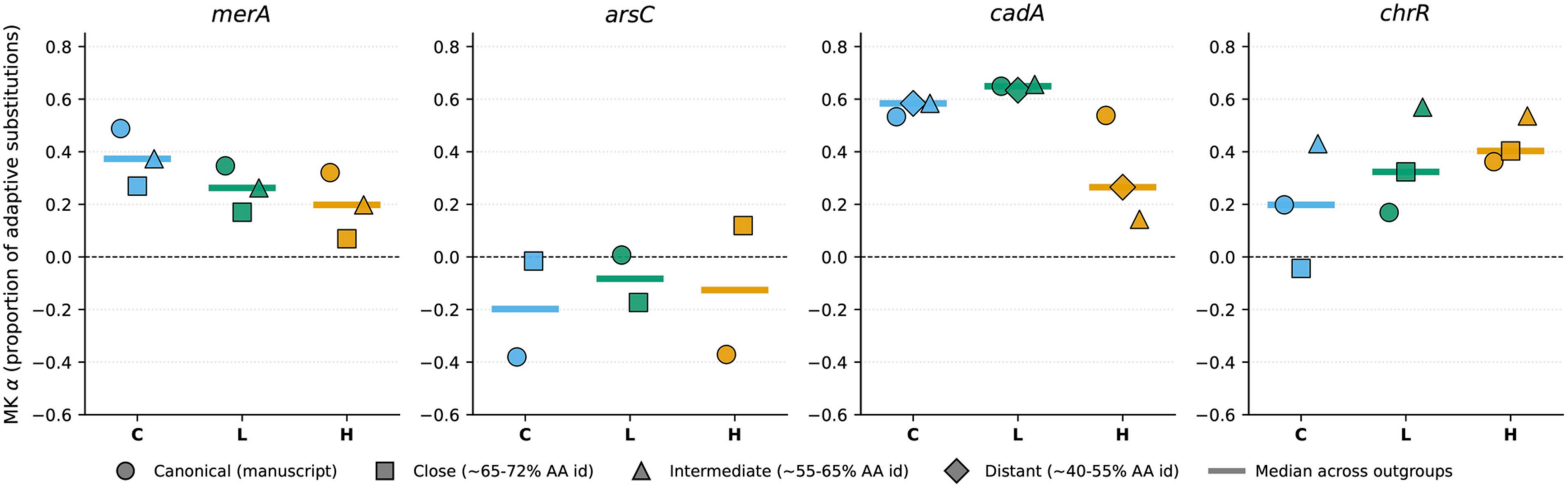
McDonald-Kreitman *α* across outgroups (MACSE codon alignment, 33 tests). Each point represents one outgroup-by-regime combination; the marker indicates outgroup class (circle = canonical, square = close ~65–72% AA identity, triangle = intermediate ~55–65%, diamond = distant ~40–55%). Horizontal bars indicate the median across outgroups for each regime. The dashed line at *α* = 0 indicates neutrality. Wong palette: control = sky blue, low = bluish green, high = orange.

The McDonald-Kreitman test, when interpreted as a descriptive index, revealed gene- and regime-specific patterns (Table 1; Supplementary Table S5): *merA* showed a significant excess of non-synonymous over synonymous divergence across all three regimes (*α* = +0.488, *p* < 0.001 in Control; *α* = +0.346, *p* = 0.004 in Low; *α* = +0.321, *p* = 0.007 in High), consistent with sustained patterns of diversification along the entire gradient; *cadA* exhibited the strongest and most consistent signal, with α reaching its maximum in Low (+0.649, *p* < 0.001) and remaining highly significant in Control (+0.533) and High (+0.538; all *p* < 0.001); *chrR* showed the most striking regime-specific pattern: a significant signal exclusively in High (*α* = +0.362, *p* = 0.038), where Cr concentrations are highest (19.73 ± 2.24 μg/L), and no significant signal in Control (*α* = +0.198, *p* = 0.33) or Low (*α* = +0.169, *p* = 0.440). By contrast, *arsC* showed no significant departure from neutrality in any regime (|*α*| < 0.4, *p* > 0.20). An outgroup-sensitivity analysis (Section 2.5; Figure 3; Supplementary Table S6) confirmed that the sign of *α* was preserved in 9 of 12 gene-by-regime cells (75%) and that statistical significance was preserved in 6 of 12 (50%) across canonical, close, and intermediate or distant outgroups, indicating that the qualitative gene-specific differences are not artifacts of outgroup choice.

### 3.4 Codon-level and gene-wide diversification signals (MEME, BUSTED, BUSTED-E)

**Figure 4:**
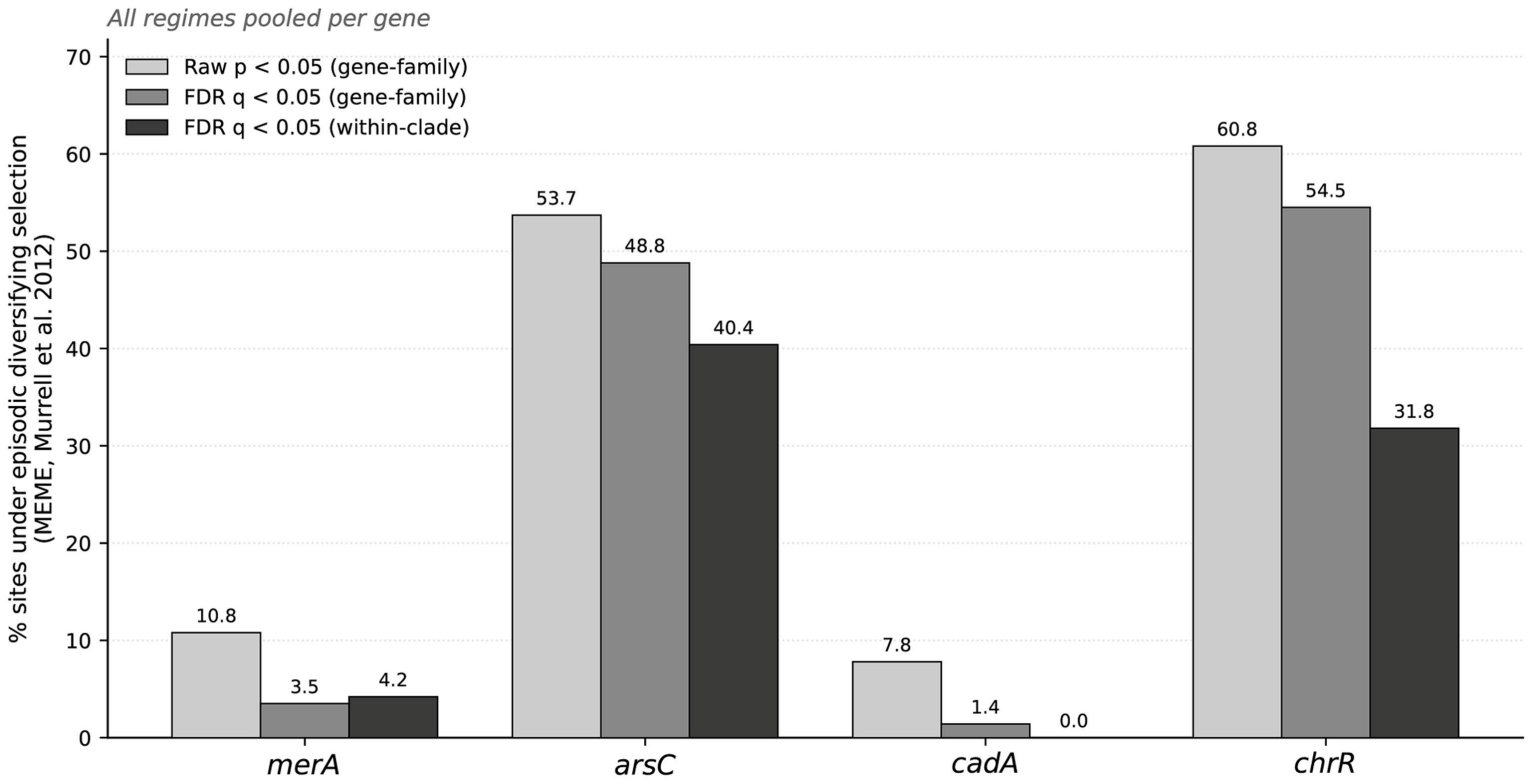
Percentage of codon sites under episodic diversifying selection (MEME, Murrell et al., 2012) per gene under three conditions: raw *p* < 0.05 at the gene-family level, Benjamini-Hochberg FDR *q* < 0.05 at the gene-family level, and FDR *q* < 0.05 within the largest MMseqs2 ortholog cluster. All values are pooled across regimes per gene.

**Figure 5:**
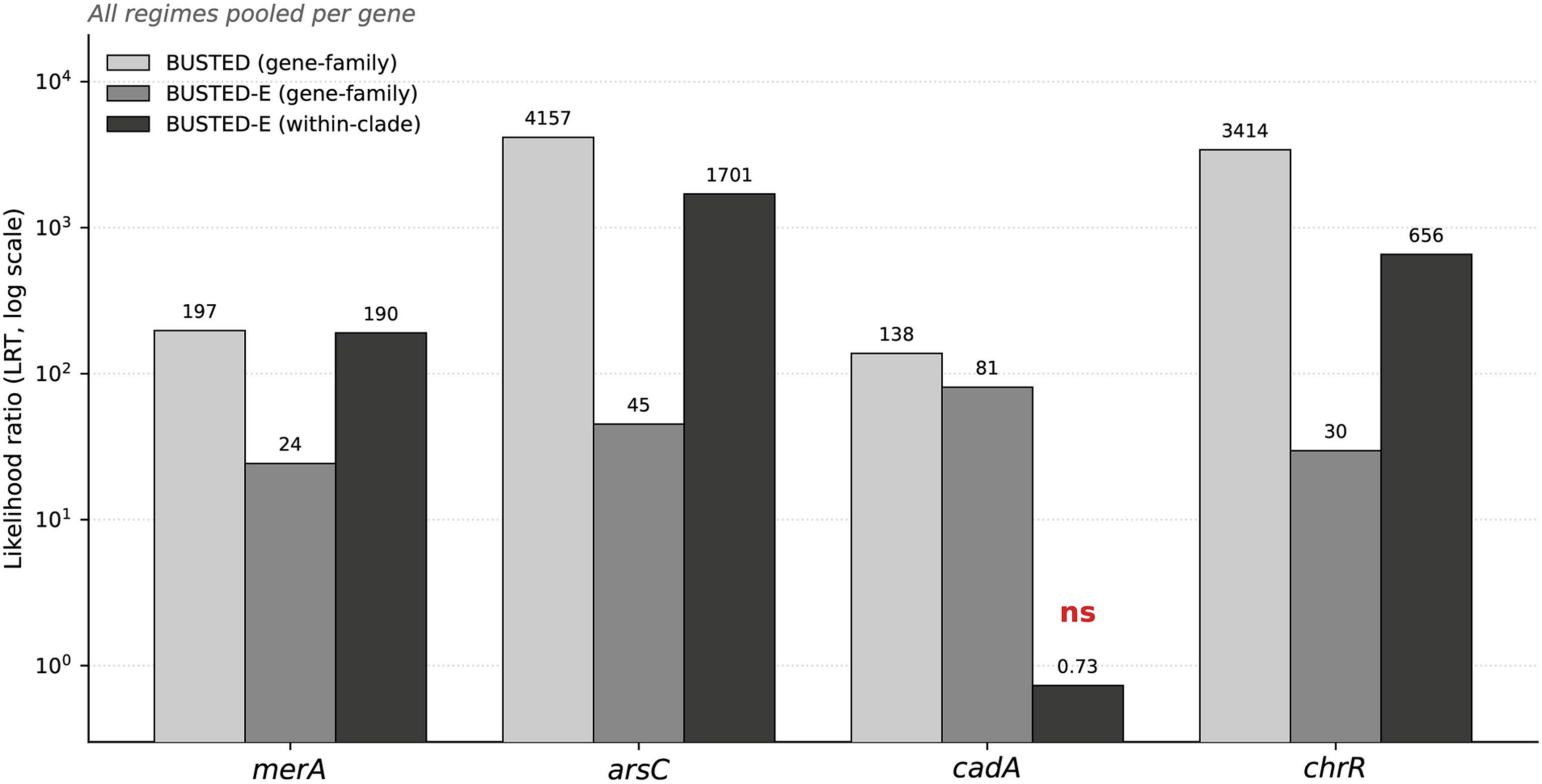
Standard BUSTED, BUSTED-E (gene-family), and BUSTED-E (within-clade) likelihood-ratio statistics (LRT, log scale) per gene. The ns annotation indicates non-significance of the BUSTED-E within-clade test for *cadA* (LRT = 0.73, *p* = 0.347). The error-sink rate class of BUSTED-E absorbed < 1% of the site weight for all four genes. All values are pooled across regimes per gene.

MEME identified codon sites with signals of episodic diversification in all four genes (Figure 4; Supplementary Table S7). After Benjamini-Hochberg FDR correction across per-codon *p*-values, the proportion of sites at *q* < 0.05 was 3.5% for *merA* (20 of 574 codons), 48.8% for *arsC* (79 of 162), 1.4% for *cadA* (10 of 727), and 54.5% for *chrR* (121 of 222). The high fractions for *arsC* and *chrR* remain implausibly large for genuine episodic selection and prompt the orthology-aware re-analysis reported in Section 3.7. BUSTED-E confirmed gene-wide patterns consistent with episodic diversification in all four genes after accounting for potential alignment errors (*merA* LRT = 24.26, *p* = 2.7 × 10^−6^; *arsC* LRT = 45.11, *p* = 8.0 × 10^−11^; *cadA* LRT = 80.82, *p* < < 0.001; *chrR* LRT = 29.69, *p* = 1.8 × 10^−7^). To assess the contribution of the error-sink rate class, we additionally ran standard BUSTED (Figure 5): the standard-BUSTED LRTs were 8 × to 115 × larger than the BUSTED-E LRTs (*merA* 197.26, *arsC* 4156.66, *cadA* 137.64, *chrR* 3414.04), confirming that BUSTED-E’s error-sink class absorbs a substantial fraction of the signal that standard BUSTED would attribute to selection in datasets with alignment heterogeneity, and that the BUSTED-E values reported here are the appropriate conservative inference for metagenomic data. The error-sink rate class absorbed less than 1% of the site weight for all four genes.

### 3.5 Phylogenetic diversity and beta-diversity (Faith’s PD and PERMANOVA)

To complement the population-genetic statistics with assumption-light community-level inference, we report Faith’s phylogenetic diversity (PD) and PERMANOVA on patristic distance matrices (Supplementary Tables S8, S9). PD was lowest for *merA* (0.74, 4.67, 7.92 across Control, Low, High) and rose monotonically with runoff; *arsC* peaked in High (33.19, 22.01, 35.53); *cadA* was maximal in Low (5.20, 7.94, 7.70); and *chrR* was highest in Control and lowest in Low (58.57, 13.73, 38.63, Supplementary Figure S4). PERMANOVA on patristic distance matrices (9,999 permutations of regime labels) showed that regime explained a small but highly significant fraction of among-haplotype distance for *merA* (*R*^2^ = 0.064, *p* < 0.001), a moderate fraction for *arsC* (*R*^2^ = 0.232, *p* < 0.001) and *cadA* (*R*^2^ = 0.154, *p* < 0.001), and a large fraction for *chrR* (*R*^2^ = 0.718, *p* < 0.001; Supplementary Figure S5). The unusually high *R*^2^ for *chrR* indicates strong phylogenetic structuring of *chrR* haplotypes by regime, which we revisit in Sections 3.7 and 3.8 considering the orthology-aware and taxonomic additional analyses.

### 3.6 Correlation between nucleotide diversity and metal concentrations

Descriptive Spearman rank correlations between regime-level *π* and target metal concentrations (Supplementary Table S10) revealed gene-specific associations (Figure 1): *cadA* π and Zn showed *ρ* = +0.50, with the highest *π* in High where Zn concentrations are highest; *arsC* and As also showed *ρ* = +0.50, reflecting a partial monotonic relationship; *merA* showed *ρ* = +0.50 with total Hg, but the relationship is shaped by the non-monotonic Hg concentration pattern (Low > High > Control) combined with the monotonic increase of *merA* diversity along the runoff gradient: *π* tracks the integrated Hg flux rather than instantaneous concentration. *chrR* showed a negative correlation with Cr (*ρ* = −0.50), driven by its non-monotonic *π* trajectory (maximum in Low, minimum in High) despite the monotonic increase of Cr. With only three regimes the Spearman *p*-values are not statistically interpretable as hypothesis tests; the Bayesian directional probabilities *P*(*π*_H_ > *π*_C_) reported in Section 3.1 provide complementary directional support.

### 3.7 Gene-family versus within-clade re-analysis

To test whether the apparent gene-specific signals above reflect within-orthogroup diversification rather than shifts in gene-family composition across regimes, we re-ran all selection statistics restricted to the largest MMseqs2 cluster of each gene (Supplementary Table S11; Supplementary Figure S6). MMseqs2 clustering at the per-gene elbow threshold (Supplementary Table S12; Supplementary Figure S7) confirmed substantial heterogeneity in orthology coherence among the four gene families: *merA* approached a single orthologous group within our dataset (90% of haplotypes in the largest cluster), whereas *cadA* was highly heterogeneous (the largest clade accounting for only 22% of haplotypes), and *arsC* (40%) and *chrR* (45%) were intermediate (Table 2). Within the largest cluster of each gene, the qualitative direction of the gene-family-level MK signal was preserved for *merA* (*α* within-clade = +0.47 / +0.50 / +0.51 for C/L/H). The *π*_N_/*π*_S_ pattern and absence of MK signal for *arsC* were also preserved (*α* within-clade = −0.37 / +0.19 / −0.16). For *merA*, BUSTED-E within-clade was strongly reinforced (LRT = 189.92 vs. gene-family 24.26) and MEME *q* < 0.05 was essentially unchanged (4.2% within-clade vs. 3.5% gene-family, FDR-corrected), reflecting its high orthology coherence and supporting the interpretation that the diversifying selection signal for *merA* is intrinsic to the dominant orthogroup.

**Table 2:** MMseqs2 ortholog clustering at the data-driven elbow threshold per gene, and selection statistics within the largest ortholog cluster (within-clade re-analysis).

Gene	Threshold	Clusters	Largest cluster	*α*(MK) C/L / H	BUSTED-E LRT	MEME % sites
*merA*	0.83	10	217/242 (90%)	+0.47 / +0.50 / +0.51	189.92 (*p* < 0.001)	4.2%
*arsC*	0.79	83	540/1,349 (40%)	−0.37 / +0.19 / −0.16	1700.95 (*p* < 0.001)	40.4%
*cadA*	0.70	15	16/74 (22%)	*n* = 0 / +0.71 / +0.58	0.73 (*p* = 0.347, ns)	0.0%
*chrR*	0.73	42	503/1,121 (45%)	+0.18 / +0.41 / *n* = 0	655.95 (*p* < 0.001)	31.8%

*α*(MK) within-clade C/L/H reports the McDonald-Kreitman proportion of adaptive substitutions per regime computed within the largest cluster. *N* = 0 indicates that the largest cluster contains no haplotypes from that regime. BUSTED-E LRT and MEME % sites (FDR q < 0.05) are gene-wide statistics pooling all regimes within the largest cluster.

By contrast, for *cadA,* the BUSTED-E signal of episodic diversifying selection at the gene-family level (LRT = 80.82) collapsed to non-significance within the largest clade (LRT = 0.73, *p* = 0.347), and the MEME percentage dropped from 1.4% (gene-family, FDR) to 0.0% (within-clade, FDR). We note that the *cadA* diversifying-selection signal at the gene-family level cannot be unambiguously assigned to within-orthogroup evolution, and is at least partly driven by heterogeneity among *cadA* subfamilies. The MK signal for *cadA*, in contrast, persists within the largest clade in the two regimes where the clade is populated (*α* = +0.71 in Low and +0.58 in High); the 19 Control *cadA* haplotypes are distributed among smaller subfamilies and do not contribute to the largest-cluster analysis. For *chrR,* the BUSTED-E signal was strongly preserved within the largest cluster (LRT = 655.95, *p* < < 0.001), and MEME % decreased from 54.5 to 31.8% after FDR correction within the clade. However, the largest *chrR* cluster was dominated by Control sequences (484 of 503, 96%); Low- and High-regime *chrR* haplotypes partitioned into other clusters, indicating that the regime contrasts in *chrR* reflect compositional turnover among orthologous subfamilies rather than within-orthogroup evolution. This interpretation is reinforced by the taxonomic profile (Section 3.8).

### 3.8 Taxonomic composition of MRG-containing haplotypes

Taxonomic affiliation of MRG-containing haplotypes, aggregated to the order level to ensure cross-gene comparability, revealed strongly gene- and regime-specific patterns (Supplementary Table S13; Supplementary Figure S8). For *merA*, we observed a monotone compositional turnover along the gradient: in Control, 77% of haplotypes were best-matched to Chromatiales; in Low, the Chromatiales fraction fell to 29% while unclassified Pseudomonadota rose to 63%; in High, the dominant matches shifted again toward Gammaproteobacteria (47%) and Pseudomonadota (33%), with the first appearance of Acidobacteriota and Actinomycetota. For *arsC*, Burkholderiales dominated in Control (50%) and High (47%), while Low was the most diversified regime (Hyphomicrobiales rising to 45%). For *cadA*, no single order dominated in any regime; Control was largely unclassified, Low was dominated by Gammaproteobacteria (21%) and Acidobacteriota (24%), and High shifted strongly toward Bacteroidota (38%, including Chitinophagaceae, *Pedobacter*, and *Chitinophaga*). Most strikingly, for *chrR*, we observed a shift from a diversified Control community (Hyphomicrobiales 80%, with a long tail of unclassified Pseudomonadota and other groups) to a near-monoculture in High (Hyphomicrobiales 93%, predominantly Xanthobacteraceae). We interpret this as strong evidence that the regime contrast for *chrR* is dominated by compositional turnover at the community level, rather than by within-orthogroup diversification consistent with the within-clade re-analysis reported in Section 3.7.

## 4 Discussion

Multiple complementary analyses converged on the conclusion that variable glacial meltwater inputs at Lake Hazen co-occur with detectable, gene- and regime-specific patterns of sequence variation in metal resistance gene pools. Because these inferences derive from metagenomic gene pools sampled across only three hydrological regimes, and because metagenomic gene pools aggregate variants across taxa and across potentially divergent members of each gene family, we interpret the patterns found here as exploratory, hypothesis-generating observations rather than as formal demonstrations of population-level adaptation. The orthology-aware (Section 3.7) and taxonomy-aware (Section 3.8) analyses are critical to this interpretation: they identify which patterns are robust at the within-orthogroup level and which depend on inter-cluster heterogeneity or community-composition shifts.

Three contrasting gene-level patterns emerged from this integrated analysis. The first sustained patterns consistent with diversification along the runoff gradient were observed for *merA*. This gene showed monotonic gene-pool diversification, a McDonald-Kreitman *α* significant across all three regimes, and these signals were preserved (indeed reinforced) within the largest orthologous cluster, which contained 90% of *merA* haplotypes. We interpret this as the strongest within-orthogroup signal in our dataset, consistent with the sustained mercury exposure associated with meltwater delivery to the lake basin and with the well-documented mobility of *merA* on the Tn21 transposon family (Liebert et al., 1999). Although Hg concentrations are highest in the Low runoff regime rather than in High, *merA* diversity tracks the runoff gradient rather than the instantaneous concentration. This suggests that the standing Hg exposure of soil microbiomes is best captured by flux-based metrics, which integrate concentration over the much greater discharge volume of the Snowgoose and Abbé river systems compared to Blister Creek (St. Pierre et al., 2019). Recent experimental work on *merA* adaptation supports this interpretation, with directed evolution and field surveys showing that Hg exposure selects for substrate-specific *merA* variants on relatively short timescales (Christakis et al., 2021).

The second pattern, regime-specific selection signals partly attributable to subfamily heterogeneity, was observed for *cadA* and *chrR*. For *cadA*, the simultaneous detection of a strong MK *α* (+0.65 in Low; +0.54 in High) and a 30% reduction in *π*_N_/*π*_S_ in High initially suggested ongoing exploration of variant space, with environmental filtering progressively narrowing the functionally permissible set. The orthology-aware analysis qualified this interpretation: the BUSTED-E signal at the gene-family level (LRT = 80.82) collapsed within the largest *cadA* cluster (LRT = 0.73, ns), and MEME % dropped from 1.4 to 0% after FDR correction within-clade. The MK signal, however, persisted within the largest clade in the two regimes where the clade was populated. We interpret this as evidence that the *cadA* diversifying signal at the gene-family level is driven in part by heterogeneity among *cadA* subfamilies, while the MK component of the signal is at least partly intrinsic to the dominant orthogroup. The biological relevance of *cadA* subfamily heterogeneity is supported by the known substrate breadth of cadA-family P-type ATPases for Zn, Cd, and Co (Argüello et al., 2007).

For *chrR*, the local MK signal restricted to the High regime, combined with the highest MEME percentage of any gene and the strongest PERMANOVA regime structure (*R*^2^ = 0.72), suggests patterns consistent with diversification in the chromium-enriched Abbé-Snowgoose river system. The orthology- and taxonomy-aware analyses qualified this interpretation: the largest *chrR* cluster was 96% Control, and the High regime was 93% Hyphomicrobiales (predominantly Xanthobacteraceae) at the community level. We therefore interpret the *chrR* regime contrast as dominated by compositional turnover, the displacement of a diversified Control *chrR* community by a Xanthobacteraceae-dominated High-runoff community rather than by within-othogroup diversification. The MEME and BUSTED-E signals that persist within the Control-dominated cluster are biologically intriguing, but should be interpreted cautiously as they describe variation within a single community context.

The third pattern, a near-absence of detectable diversifying signal, was observed for *arsC*. The MK *α* was non-significant or negative in all three regimes, and these patterns were preserved within the largest orthologous cluster. A biogeochemical consideration is that As (a metalloid) and Cr (a metal) are redox-sensitive elements that occur in the environment primarily as oxyanions (arsenate, arsenite, chromate), whose mobility, bioavailability, and cellular uptake depend strongly on local pH and redox potential. Their cellular accessibility, therefore, differs from that of divalent cations such as Zn^2+^, Cd^2+^, and Hg^2+^, which form cationic ligand complexes and follow different transport pathways into the cell. On this basis, one might expect *arsC* and *chrR*, which both target oxyanion substrates, to behave similarly. Our data do not support that simple grouping: even after the orthology- and taxonomy-aware controls, *chrR* shows the strongest community-level regime structure of the four genes, whereas *arsC* shows no detectable diversifying signal in any regime. The contrast indicates that substrate speciation alone is not sufficient to predict the evolutionary trajectory of an MRG in this system, and that gene-specific factors, including the magnitude of the local exposure gradient, host-cell physiology, and the operon context in which each gene is embedded, must also be considered.

Several methodological considerations should be put forward. First, metagenomic gene pools aggregate sequence variants across taxa and across potentially divergent members of each gene family. The polymorphism/divergence partition required by the McDonald-Kreitman framework, and the panmictic-population assumption underlying Tajima’s *D*, are not strictly satisfied in this setting (Ellegaard and Engel, 2016; Garud and Pollard, 2020). Accordingly, the McDonald-Kreitman, MEME, and BUSTED/BUSTED-E results reported here should be read as descriptive, hypothesis-generating summaries rather than as formal evidence of selection, because the assumptions of population coherence, strict orthology, and vertical inheritance on which these tests rest are only partially satisfied in metagenomic gene pools. Consistent with this, some MEME and BUSTED-E signals remain unexpectedly high even after orthology and taxonomy controls; residual alignment heterogeneity, horizontal gene transfer, recombination, and incompletely resolved ortholog structure can each inflate apparent episodic-selection signals in metagenomic alignments and likely contribute to these residuals. We addressed this directly by presenting all population-genetic statistics as descriptive indices, by clustering sequences into orthologous subfamilies,. We also performed selection analyses within the largest cluster, by applying FDR correction across all per-codon and per-test outputs, and by running outgroup-sensitivity analyses for the MK test. These within-clade analyses (Section 3.7) showed that the *merA* pattern is robust, the *arsC* absence of a positive signal is robust, the *cadA* BUSTED-E signal disappears (indicating subfamily heterogeneity), and the *chrR* regime structure reflects compositional turnover. Second, coalescent estimators of *N*_e_ assume neutrality (Charlesworth, 2009) and tree-like vertical inheritance (Soucy et al., 2015). For these reasons, BEAST2 and MASCOT outputs are interpreted only as descriptive gene-pool diversity and exchange indices (Tables S14 and S15). Third, horizontal gene transfer of MRGs via plasmids and transposons (Liebert et al., 1999; Silver and Phung, 1996) means that contemporary *merA*, *cadA* haplotypes may reflect recent transfer events rather than long-term within-lineage evolution: this constrains the timescales over which selection signals can be interpreted. The convergence of orthology-aware, taxonomy-aware, and outgroup-aware analyses on a coherent gene-by-gene story (Sections 3.7–3.8) is what permits the above interpretions.

The patterns documented here have practical implications for monitoring Arctic ecosystems under accelerating climate change. The orthology-robust *merA* pattern indicates that Hg-resistance gene pools can respond on timescales relevant to contemporary glacial retreat and could serve as candidate sensitive biomarkers complementing traditional geochemical measurements. The *chrR* compositional turnover finding indicates that Cr exposure is associated with community-level changes that can themselves be informative. The *cadA* and *arsC* findings caution that gene-family-level statistics should not be over-interpreted in metagenomic datasets without orthology controls.

Several limitations should be acknowledged. First, the analysis covers a single watershed; replicating the space-for-time substitution at other Arctic sites would test generality. Second, the small number of *cadA* haplotypes (*n* = 19 to 29 per regime) limits the precision of estimates for this gene. Third, in-situ metal speciation and redox conditions were not measured, and our interpretation is therefore restricted to total metal pools across regimes. Fourth, taxonomic affiliation could not be determined for 76 *chrR* Low-regime haplotypes due to computational wall-clock constraints (92% overall taxonomic coverage). Their omission does not affect the Control vs. High contrast that drives our interpretation, but should be addressed in future work. Fifth, the orthology controls used here (MMseqs2 elbow-threshold clustering) define operational orthogroups by sequence-identity neighborhood rather than by formal phylogenetic orthology inference; sensitivity to alternative orthology definitions is reported in Supplementary Table S12. Sixth, functional validation of regime-specific variants (particularly *chrR* variants in the High regime) was not attempted in this study, but would be essential for testing the phenotypic significance of the genetic signatures inferred here. Long-read metagenomics combined with genome binning would clarify the contribution of host phylogeny vs. mobile element dynamics. As climate-driven mobilization of previously sequestered metals accelerates across the Arctic, monitoring the diversification and sequence variation in MRG gene pools alongside chemical measurements may provide an integrative framework for assessing the biological consequences of these rapid environmental changes.

## Data Availability

Publicly available datasets were analyzed in this study. This data can be found: The raw metagenomic sequencing reads analyzed in this study are publicly available in the NCBI Sequence Read Archive under BioProjects PRJNA556841 (Colby et al., 2020). The trace metal and total mercury concentration data are from St. Pierre et al. (2019). All custom analysis scripts, used in this study are available in the GitHub repository https://github.com/Franckoued/mrg-evolution-lakehazen.git.
