# Supplementary material for: Glacial meltwater is associated with gene-specific diversification of metal resistance genes in high Arctic soil microbiomes
Source: Front Microbiol. 2026 Jul 27;17:1903619. doi: 10.3389/fmicb.2026.1903619 (PMC13454099; doi:10.3389/fmicb.2026.1903619)
Supplement: Supplementary file 1 [file Data_Sheet_1.pdf]

Supplementary Material

Glacial meltwater is associated with gene-specific diversification of metal resistance genes in High Arctic soil microbiomes

Franck J. Ouedraogo, Alexandre J. Poulain, Stéphane Aris-Brosou

Department of Biology, University of Ottawa, Ottawa, Ontario, Canada

Contents

Supplementary Tables (S1–S15): S1 (π per site), S2 (Tajima's D), S3 (rarefaction/subsampling), S4 (π_N/π_S), S5 (McDonald-Kreitman), S6 (MK outgroup sensitivity MACSE), S7 (MEME), S8 (Faith's PD), S9 (PERMANOVA), S10 (metal concentrations), S11 (within-clade selection statistics), S12 (MMseqs2 clustering at multiple thresholds), S13 (taxonomic composition order-level), S14 (BEAST2 θ), S15 (MASCOT migration rates).

Supplementary Figures (S1–S8): S1 (analysis workflow), S2 (Tajima's D), S3 (π_N/π_S), S4 (Faith's PD), S5 (PERMANOVA R^2), S6 (gene-family vs within-clade), S7 (MMseqs2 ortholog cluster summary), S8 (taxonomic composition order-level).

1 Supplementary Tables

Table S1

Table S1. Nucleotide diversity (π) per gene and individual sampling site, computed by the Nei pairwise method with 500 bootstrap replicates for 95% confidence intervals. Sites grouped by hydrological regime and sample type (SED = sediment, SOIL = soil). Missing values indicate insufficient sequence recovery (N < 5).

Gene	Site	Regime	Type	N	π [95% CI]
merA	CSOIL	C	Soil	60	0.0444 [0.0359–0.0538]
	LSOIL	L	Soil	99	0.0691 [0.0471–0.0965]
	H1	H	Sed	112	0.1840 [0.1634–0.2039]
	HSOIL	H	Soil	83	0.0888 [0.0636–0.1247]
arsC	CSED	C	Sed	10	0.3347 [0.2474–0.3876]
	CSOIL	C	Soil	550	0.1903 [0.1814–0.1997]
	L1	L	Sed	56	0.3193 [0.2893–0.3400]
	L2	L	Sed	135	0.3092 [0.2957–0.3246]
	LSOIL	L	Soil	276	0.2372 [0.2233–0.2500]
	H1	H	Sed	238	0.2522 [0.2421–0.2621]
	H2	H	Sed	71	0.2750 [0.2477–0.2989]
	HSOIL	H	Soil	523	0.2028 [0.1935–0.2109]
cadA	CSOIL	C	Soil	19	0.3103 [0.1979–0.3891]
	L1	L	Sed	6	0.2620 [0.1332–0.3444]
	L2	L	Sed	10	0.2463 [0.0946–0.3518]
	LSOIL	L	Soil	29	0.2962 [0.2101–0.3701]
	H1	H	Sed	15	0.3559 [0.2608–0.4066]
	H2	H	Sed	5	0.2924 [0.0040–0.4132]
	HSOIL	H	Soil	26	0.4234 [0.3939–0.4414]
chrR	CSED	C	Sed	73	0.1223 [0.1053–0.1388]

CSOIL	C	Soil	726	0.1499 [0.1433–0.1567]
L1	L	Sed	44	0.2317 [0.1879–0.2676]
L2	L	Sed	217	0.1501 [0.1362–0.1658]
LSOIL	L	Soil	76	0.2399 [0.2178–0.2646]
H1	H	Sed	327	0.1999 [0.1826–0.2163]
H2	H	Sed	150	0.2397 [0.2186–0.2610]
HSOIL	H	Soil	319	0.1301 [0.1182–0.1426]

Table S2

Table S2. Tajima's D statistics for soil-only haplotypes with significance levels. * $p < 0.05$, ** $p < 0.01$, *** $p < 0.001$. Negative D values may indicate population expansion or purifying selection; positive values may indicate contraction or balancing selection.

Gene	Regime	N haplotypes	Tajima's *D*	*p*-value	Significance
merA	Control	60	−1.065	n.s.	ns
	Low	99	−1.675	< 0.05	*
	High	83	−1.630	< 0.05	*
arsC	Control	550	+1.077	n.s.	ns
	Low	276	+1.900	< 0.05	*
	High	523	+1.353	n.s.	ns
cadA	Control	19	+0.244	n.s.	ns
	Low	29	+0.187	n.s.	ns
	High	26	−0.167	n.s.	ns
chrR	Control	726	+0.199	n.s.	ns
	Low	76	+0.785	n.s.	ns
	High	319	−0.417	n.s.	ns

Table S3

Table S3. Rarefaction/subsampling of π to a common haplotype depth (n = 19, the smallest gene-by-regime sample; *cadA* Control). For each gene-by-regime cell, we report π computed on the dereplicated full data, the subsampled π mean and 95% bootstrap CI from 500 replicates of size n = 19, and whether the rank direction relative to the previous regime is preserved.

Gene	Regime	n full	π (full)	π (subsampled) [95% CI]	Rank direction preserved
merA	C	60	0.044	0.044 [0.033–0.057]	-
	L	99	0.069	0.068 [0.048–0.094]	C < L (Yes)
	H	83	0.089	0.088 [0.062–0.122]	L < H (Yes)
arsC	C	550	0.190	0.188 [0.169–0.209]	-
	L	276	0.237	0.236 [0.213–0.260]	C < L (Yes)
	H	523	0.203	0.202 [0.183–0.223]	L > H (Yes)
cadA	C	19	0.310	0.310 [0.198–0.389]	-
	L	29	0.296	0.288 [0.180–0.376]	C ~ L (overlap)
	H	26	0.423	0.420 [0.388–0.443]	L < H (Yes)
chrR	C	726	0.150	0.149 [0.139–0.160]	-
	L	76	0.240	0.239 [0.215–0.267]	C < L (Yes)
	H	319	0.130	0.130 [0.117–0.146]	L > H (Yes)

Table S4

Table S4. Nucleotide diversity at non-synonymous (π_N) and synonymous (π_S) sites computed using the Nei-Gojobori (1986) method, with 95% bootstrap confidence intervals (50 resamples) on the π_N/π_S ratio. Ratios below 1 indicate purifying selection. Independent of N_e estimation.

Gene	Regime	π_N	π_S	π_N/π_S	95% CI	# codons
merA	Control	0.0181	0.0825	0.220	[0.192–0.237]	478
	Low	0.0212	0.0867	0.245	[0.210–0.277]	478
	High	0.0288	0.1082	0.266	[0.228–0.323]	478
arsC	Control	0.0805	0.2386	0.337	[0.325–0.348]	142
	Low	0.1078	0.2403	0.449	[0.421–0.476]	142
	High	0.0939	0.2079	0.452	[0.432–0.467]	144
cadA	Control	0.1024	0.1182	0.866	[0.804–0.982]	462
	Low	0.1185	0.1384	0.856	[0.757–0.903]	389
	High	0.0802	0.1322	0.607	[0.521–0.699]	627
chrR	Control	0.0667	0.1158	0.576	[0.554–0.589]	194
	Low	0.0962	0.2117	0.454	[0.422–0.496]	190
	High	0.0530	0.0843	0.630	[0.596–0.661]	196

Table S5

Table S5. McDonald-Kreitman test (1991) results per gene and regime. P_N/P_S: intra-regime polymorphisms. D_N/D_S: fixed differences vs outgroup. NI = $(P_N/P_S)/(D_N/D_S)$. $\alpha = 1 - \text{NI}$ (Smith and Eyre-Walker, 2002) = proportion of adaptive substitutions. Significance from χ^2 test with Yates correction (*** $p < 0.001$, ** $p < 0.01$, * $p < 0.05$, *ns* not significant). Independent of N_e estimation.

Gene	Regime	PN	PS	DN	DS	χ²	*p*	NI	α	Sig.
merA	Control	123	206	168	144	16.85	< 0.0001	0.512	+0.488	***
	Low	221	319	159	150	8.39	0.0038	0.654	+0.346	**
	High	295	373	163	140	7.38	0.0066	0.679	+0.321	**
arsC	Control	130	140	37	55	1.43	0.2314	1.380	−0.380	ns
	Low	126	139	42	46	0.00	1.0000	0.993	+0.007	ns
	High	125	138	37	56	1.36	0.2429	1.371	−0.371	ns
cadA	Control	289	274	278	123	30.57	< 0.0001	0.467	+0.533	***
	Low	316	323	251	90	52.22	< 0.0001	0.351	+0.649	***
	High	394	473	330	183	45.33	< 0.0001	0.462	+0.538	***
chrR	Control	167	176	71	60	0.94	0.3319	0.802	+0.198	ns
	Low	153	153	71	59	0.60	0.4370	0.831	+0.169	ns
	High	138	141	89	58	4.32	0.0378	0.638	+0.362	*

Table S6

Table S6. Outgroup sensitivity of the McDonald-Kreitman α using MACSE codon alignment (Ranwez et al., 2018). For each gene, we report α per regime computed with the canonical outgroup (manuscript original) and with one to two additional BLAST-derived outgroups in distinct amino-acid identity bins: close (~65–72%), intermediate (~55–65%), and distant (~40–55%). Across the 33 outgroup-by-regime cells, the sign of α was preserved in 9 of 12 gene-by-regime combinations (75%), and statistical significance at $p < 0.05$ was preserved in 6 of 12 (50%).

Gene	Regime	Canonical α	Close α	Intermediate α	Distant α	Sign preserved
merA	C	+0.488	+0.269	+0.373	-	Yes
	L	+0.346	+0.170	+0.262	-	Yes
	H	+0.321	+0.069	+0.198	-	Yes
arsC	C	−0.380	−0.016	-	-	Yes (negative)
	L	+0.007	−0.173	-	-	Mixed
	H	−0.371	+0.119	-	-	No
cadA	C	+0.533	-	+0.584	+0.584	Yes
	L	+0.649	-	+0.656	+0.634	Yes
	H	+0.538	-	+0.143	+0.265	Yes
chrR	C	+0.198	−0.043	+0.431	-	Mixed
	L	+0.169	+0.323	+0.569	-	Yes
	H	+0.362	+0.403	+0.536	-	Yes

Table S7

Table S7. Detailed MEME results per gene at the gene-family level: number and percentage of codons under episodic positive selection at three thresholds (raw $p < 0.1$, raw $p < 0.05$, and Benjamini-Hochberg FDR-corrected $q < 0.05$). The FDR-corrected fractions are the values reported in the main text and visualized in Figure 4.

Gene	Total codons	Raw *p*<0.1 (n, %)	Raw *p*<0.05 (n, %)	FDR q<0.05 (n, %)
merA	574	48 (8.4%)	23 (4.0%)	20 (3.5%)
arsC	162	86 (53.1%)	41 (25.3%)	79 (48.8%)
cadA	727	41 (5.6%)	12 (1.7%)	10 (1.4%)
chrR	222	125 (56.3%)	67 (30.2%)	121 (54.5%)

Table S8

Table S8. Faith's phylogenetic diversity (PD) per gene and regime, computed as the total branch length of the soil-only IQ-TREE phylogeny (substitutions per site).

Gene	Regime	N haplotypes	Faith's PD (subs/site)
merA	Control	60	0.7400
	Low	99	4.6690
	High	83	7.9180
arsC	Control	550	33.1910
	Low	276	22.0060
	High	523	35.5330
cadA	Control	19	5.1950
	Low	29	7.9410
	High	26	7.7010
chrR	Control	726	58.5650
	Low	76	13.7260
	High	319	38.6300

Table S9

Table S9. PERMANOVA on patristic distance matrices from the gene-specific IQ-TREE phylogenies, testing the effect of hydrological regime on phylogenetic structure (9,999 permutations of regime labels). R^2 is the proportion of variance explained by the regime.

Gene	R^2	p-value	Permutations	Effect interpretation
merA	0.064	< 0.001	9999	Weak (< 0.2)
arsC	0.232	< 0.001	9999	Moderate (0.2–0.5)
cadA	0.154	< 0.001	9999	Weak (< 0.2)
chrR	0.718	< 0.001	9999	Strong (> 0.5)

Table S10

Table S10. Metal concentrations across hydrological regimes at Lake Hazen (mean ± SD). All values from St. Pierre et al. (2019). Control = Lake Hazen surface water and Ruggles River outflow; Low = Blister Creek; High = pooled Snowgoose + Abbé Rivers. Note that THg follows a non-monotonic pattern with maximum in Low, while Cr and Zn show monotonic increases along the runoff gradient. H/C fold-change = ratio of concentration in High vs Control regime; n.d. = not determinable (Control below detection limit).

Metal	Control	Low (Blister)	High (SG + Abbé)	Unit	H/C fold-change
THg	0.42	17.56 ± 14.14	7.07 ± 1.27	ng/L	16.6×
Cr	0.33	6.15 ± 4.92	19.73 ± 2.24	µg/L	60.2×
As	< 0.5	1.16 ± 0.98	2.69 ± 0.31	µg/L	n.d.
Zn	< 0.5	44.77 ± 46.98	108.07 ± 12.02	µg/L	n.d.

Table S11

Table S11. Within-clade selection statistics. For each gene, all selection analyses were repeated, restricted to the largest MMseqs2 cluster at the elbow threshold (Table S12). π within-clade, Tajima's D within-clade and α(MK) within-clade are reported per regime; BUSTED-E LRT and MEME % sites (FDR $q < 0.05$) are gene-wide and pool all regimes within the largest cluster. $n = 0$ indicates that the largest cluster contains no haplotypes from that regime; n.c. = not computable due to insufficient sample size.

Gene	π within-clade C / L / H	Tajima's D within-clade	α(MK) within-clade C / L / H	BUSTED-E LRT	MEME % (FDR q < 0.05)
merA	0.047 / 0.037 / 0.050	−0.78 / −1.43 / −1.40	+0.47 / +0.50 / +0.51	189.92 (p < 0.001)	4.2% (20 / 478)
arsC	0.166 / 0.149 / 0.158	+0.66 / +0.71 / +0.81	−0.37 / +0.19 / −0.16	1700.95 (p < 0.001)	40.4% (57 / 141)
cadA	n = 0 / 0.065 / 0.243	n = 0 / n.c. / n.c.	n = 0 / +0.71 / +0.58	0.73 (p = 0.347, ns)	0.0% (0 / 383)
chrR	0.156 / 0.118 / n = 0	n.c. / n.c. / n = 0	+0.18 / +0.41 / n = 0	655.95 (p < 0.001)	31.8% (62 / 195)

Table S12

Table S12. MMseqs2 ortholog clustering of MRG haplotypes at multiple sequence-identity thresholds. For each gene, we report the number of clusters and the size of the largest cluster at three reference thresholds (50%, 70%, 90% sequence identity) and at the data-driven elbow threshold determined from the per-gene identity distribution. The elbow threshold is the value retained for the within-clade re-analyses reported in the main text (Section 3.7).

Gene	Identity threshold	Clusters	Largest cluster (n, %)
merA	0.50	3	238 / 242 (98.3%)
	0.70	5	230 / 242 (95.0%)
	0.83 (elbow)	10	217 / 242 (89.7%)
	0.90	18	189 / 242 (78.1%)
arsC	0.50	12	1198 / 1349 (88.8%)
	0.70	39	858 / 1349 (63.6%)
	0.79 (elbow)	83	540 / 1349 (40.0%)
	0.90	201	232 / 1349 (17.2%)
cadA	0.50	4	61 / 74 (82.4%)
	0.70 (elbow)	15	16 / 74 (21.6%)
	0.90	42	5 / 74 (6.8%)
chrR	0.50	5	1054 / 1121 (94.0%)
	0.73 (elbow)	42	503 / 1121 (44.9%)
	0.90	187	143 / 1121 (12.8%)

Table S13

Table S13. Taxonomic composition of MRG-containing haplotypes per gene and regime, aggregated to the order level. Each cell reports the percentage of haplotypes affiliated with the given taxonomic order in the given regime, based on best-hit BLASTP against NCBI nr (E-value < 10^{-20}). (-) indicates < 1% representation or absence. *chrR* Low: not assigned (76 haplotypes not analyzed due to wall-clock constraints; see Methods Section 2.7).

merA (order-level affiliation):

Order	Control (n=60)	Low (n=99)	High (n=83)
Chromatiales	77%	29%	16%
Pseudomonadota (other)	17%	63%	33%
Gammaproteobacteria (other)	7%	8%	47%
Acidobacteriota	-	-	4%
Actinomycetota	-	-	1%

arsC (order-level affiliation):

Order	Control (n=550)	Low (n=180)	High (n=523)
Burkholderiales	50%	28%	47%
Hyphomicrobiales	17%	45%	6%
Rhodospirillales	12%	-	38%
Sphingomonadales	3%	5%	5%
Unclassified	9%	7%	-
Other	9%	15%	4%

cadA (order-level affiliation):

Order	Control (n=19)	Low (n=29)	High (n=26)
Unclassified	47%	-	12%

Acidobacteriota	16%	24%	15%
Actinomycetota	16%	7%	4%
Bacteroidota	16%	3%	38%
Gammaproteobacteria (other)	-	21%	-
Hyphomicrobiales	-	21%	-
Verrucomicrobiota	-	7%	-
Chloroflexota	-	-	8%
Other	5%	17%	23%

chrR (order-level affiliation; Low not assigned):

Order	Control (n=726)	High (n=302)
Hyphomicrobiales	80%	93%
Pseudomonadota (other)	18%	-
Alphaproteobacteria (other)	-	5%
Rhodospirillales	-	1%
Other	1%	1%

Table S14

Table S14. Detailed BEAST2 posterior estimates of gene-pool diversity index ($\theta = 2N_e\mu$) per gene and regime, with 95% highest posterior density (HPD) interval and CI width. Interpreted as a descriptive index of gene-pool diversity rather than as an estimate of organismal effective population size (see Methods Section 2.8).

Gene	Regime	N haps	θ median	95% HPD lower	95% HPD upper	CI width
merA	Control	60	0.1080	0.0780	0.1400	0.0620
	Low	99	0.2410	0.1890	0.2950	0.1060
	High	83	0.3380	0.2610	0.4190	0.1580
arsC	Control	550	1.4800	1.1590	1.8150	0.6560
	Low	276	1.5430	1.2210	1.8830	0.6620
	High	523	1.5410	1.1980	1.9030	0.7050
cadA	Control	19	0.4270	0.2400	0.6510	0.4110
	Low	29	1.1290	0.6990	1.6280	0.9290
	High	26	0.9980	0.6230	1.4190	0.7960
chrR	Control	726	1.7880	1.3860	2.2110	0.8250
	Low	76	1.1670	0.8740	1.4550	0.5810
	High	319	2.4170	1.8140	2.9670	1.1530

Table S15

Table S15. Detailed MASCOT structured coalescent inter-regime migration rates per gene, with 95% HPD intervals. Migration rates are in events per lineage per coalescent time unit. Interpreted as a descriptive index of gene-pool exchange rather than as an estimate of organismal demographic migration (see Methods Section 2.8).

Gene	From	To	Median rate	95% HPD lower	95% HPD upper
merA	C	L	0.632	0.000	1.981
	L	C	1.146	0.101	2.405
	C	H	0.528	0.000	1.662
	H	C	0.904	0.001	1.921
	L	H	0.464	0.000	1.449
	H	L	0.624	0.000	1.571
arsC	C	L	0.324	0.000	0.819
	L	C	0.174	0.000	0.546
	C	H	0.437	0.000	1.044
	H	C	0.188	0.000	0.593
	L	H	0.179	0.000	0.561
	H	L	0.259	0.000	0.657
cadA	C	L	1.306	0.000	3.427
	L	C	1.448	0.000	3.410
	C	H	1.219	0.000	3.132
	H	C	1.132	0.000	3.141
	L	H	1.109	0.000	2.894
	H	L	0.824	0.000	2.709
chrR	C	L	0.268	0.000	0.629
	L	C	0.139	0.000	0.428
	C	H	0.123	0.000	0.368

	H	C	0.106	0.000	0.319
	L	H	0.168	0.000	0.490
	H	L	0.310	0.012	0.701

2 Supplementary Figures

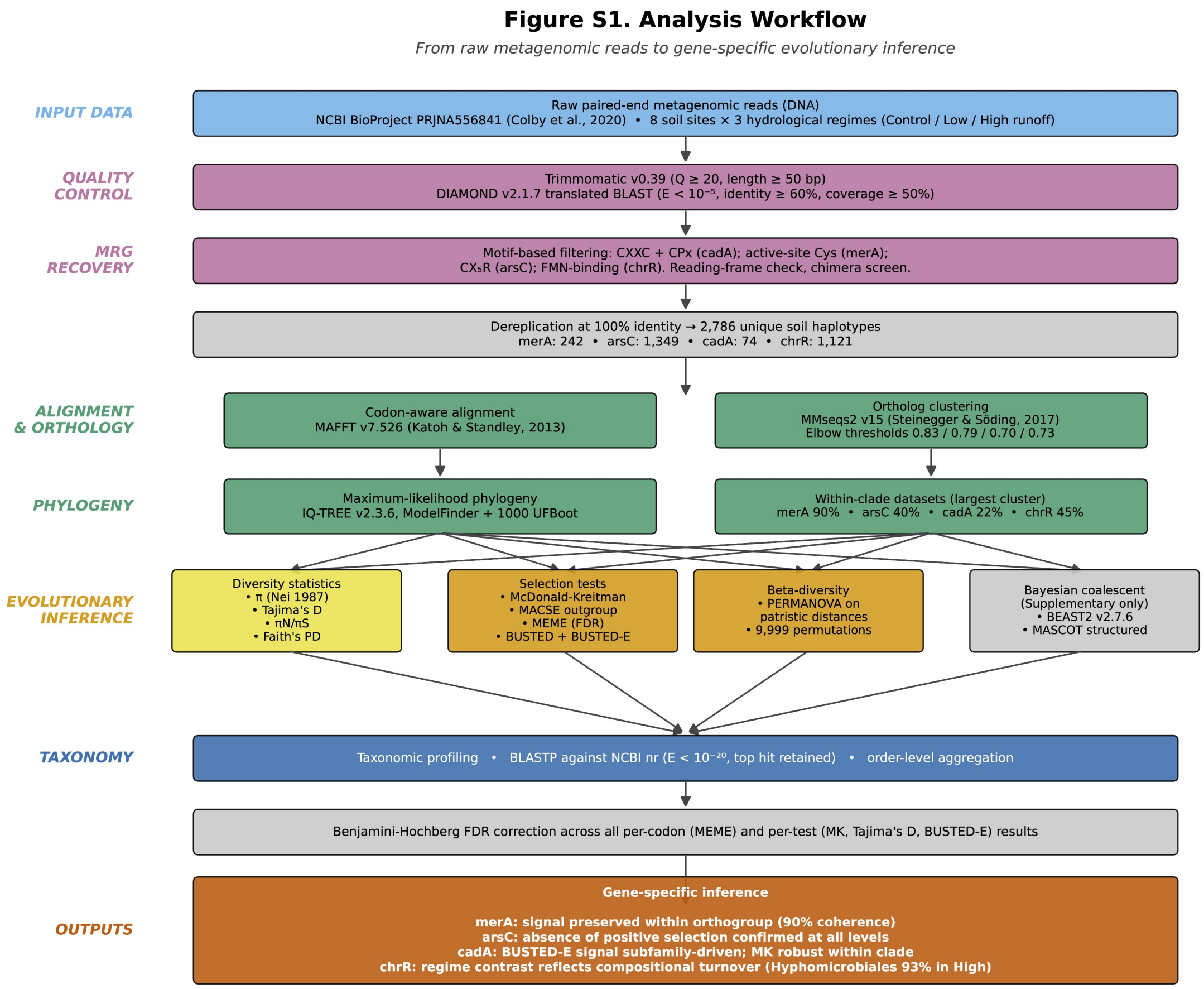

Figure S1. Analysis workflow diagram. Schematic representation of the bioinformatic and statistical pipeline used in this study, from raw metagenomic DNA reads (NCBI BioProject PRJNA556841) through quality control, MRG recovery via translated BLAST and motif filtering, dereplication, codon-aware alignment with MAFFT, ortholog clustering with MMseqs2, phylogenetic inference with IQ-TREE, population-genetic statistics (π, Tajima's D, π_N/π_S, McDonald-Kreitman), codon-based selection inference (MEME, BUSTED, BUSTED-E), phylogenetic beta-diversity (Faith's PD, PERMANOVA), gene-pool diversity and exchange (BEAST2, MASCOT), and taxonomic profiling (BLASTP against NCBI nr).

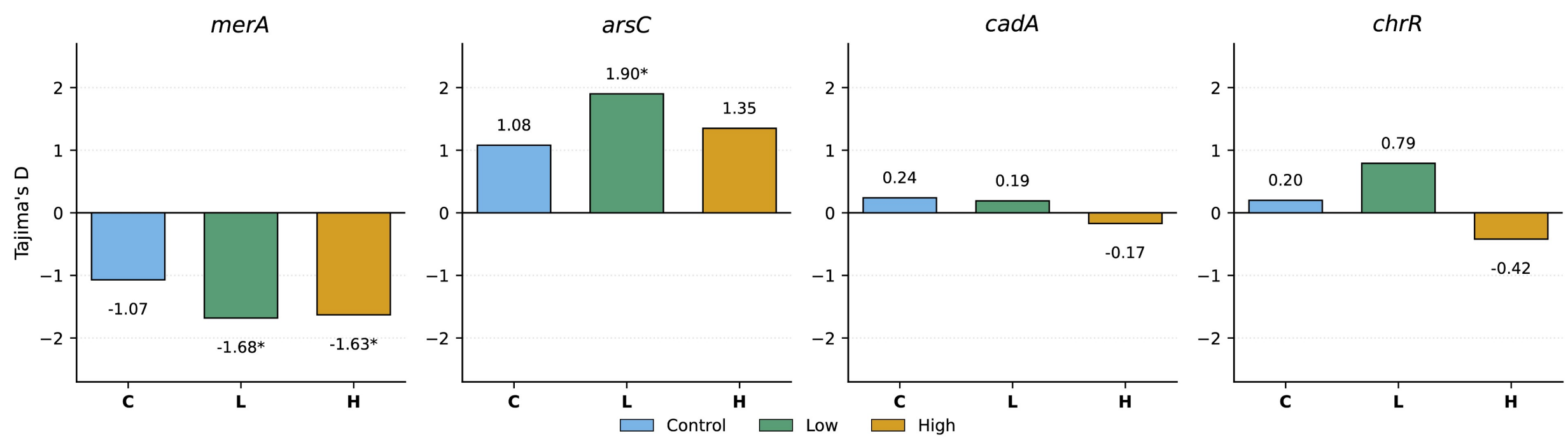

Figure S2. Tajima's *D* per gene and regime, computed on soil-only haplotypes. Bars: regime mean; numeric labels: D value with significance asterisks (* $p < 0.05$). The horizontal dashed lines at $D = \pm 2$ indicate the conventional thresholds for departure from neutrality. Wong palette: Control = sky blue, Low = bluish green, High = orange. Numerical values are reported in Table S2.

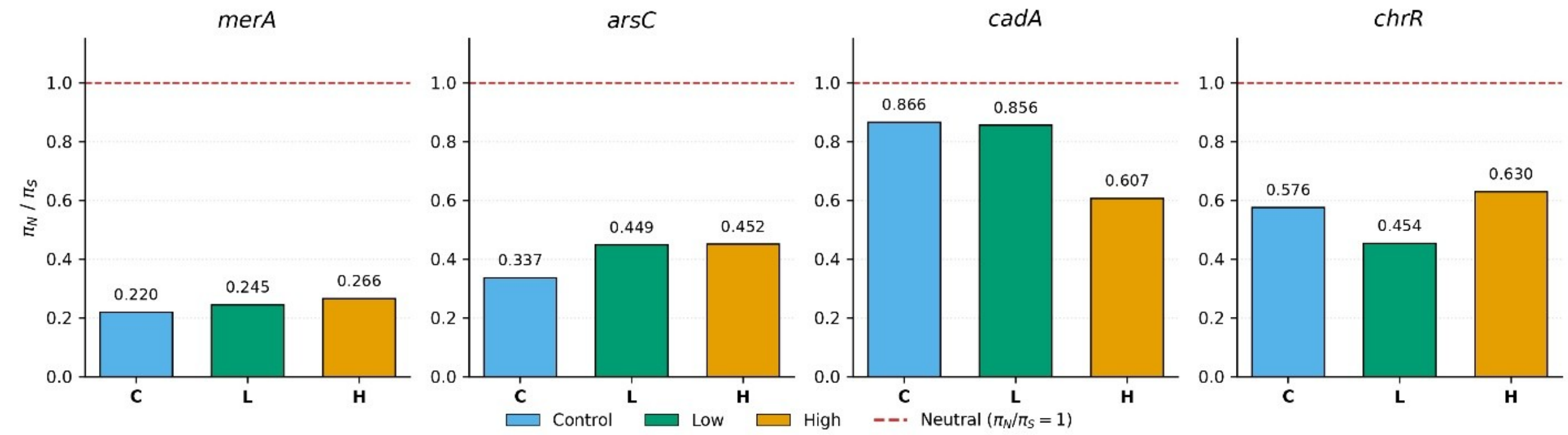

Figure S3. Non-synonymous to synonymous nucleotide diversity ratio (π_N/π_S) per gene and regime, computed with the Nei-Gojobori (1986) method. Error bars: 95% bootstrap confidence intervals (50 resamples). The red dashed line at $\pi_N/\pi_S = 1$ indicates neutrality; values below 1 indicate purifying selection. Wong palette: Control = sky blue, Low = bluish green, High = orange. Numerical values are reported in Table S4.

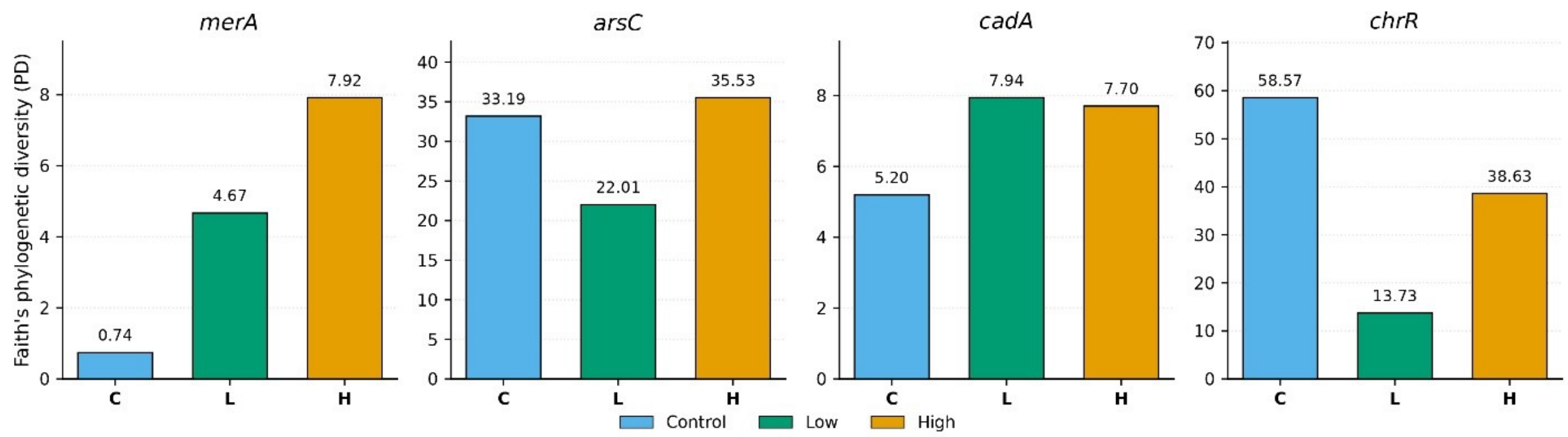

Figure S4. Faith's phylogenetic diversity (PD) per gene and regime, computed as the total branch length of the soil-only IQ-TREE phylogeny (substitutions per site). Bars: regime PD; numeric labels: PD value. Wong palette: Control = sky blue, Low = bluish green, High = orange. Numerical values are reported in Table S8.

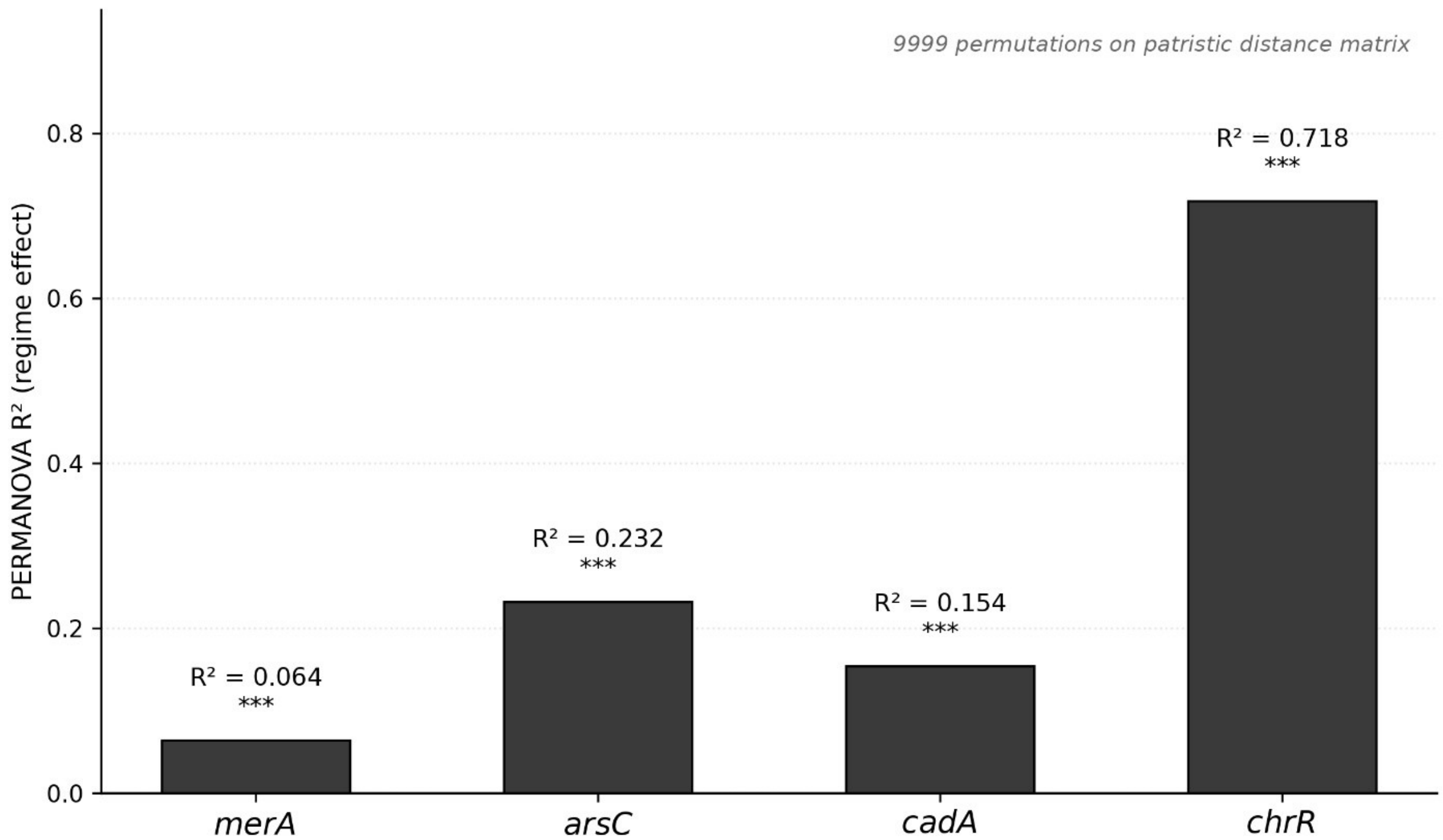

Figure S5. PERMANOVA R^2 (proportion of variance explained by regime) per gene, with significance from 9,999 permutations of regime labels on patristic distance matrices computed from the IQ-TREE phylogeny. Significance: *** $p < 0.001$. Numerical values are reported in Table S9.

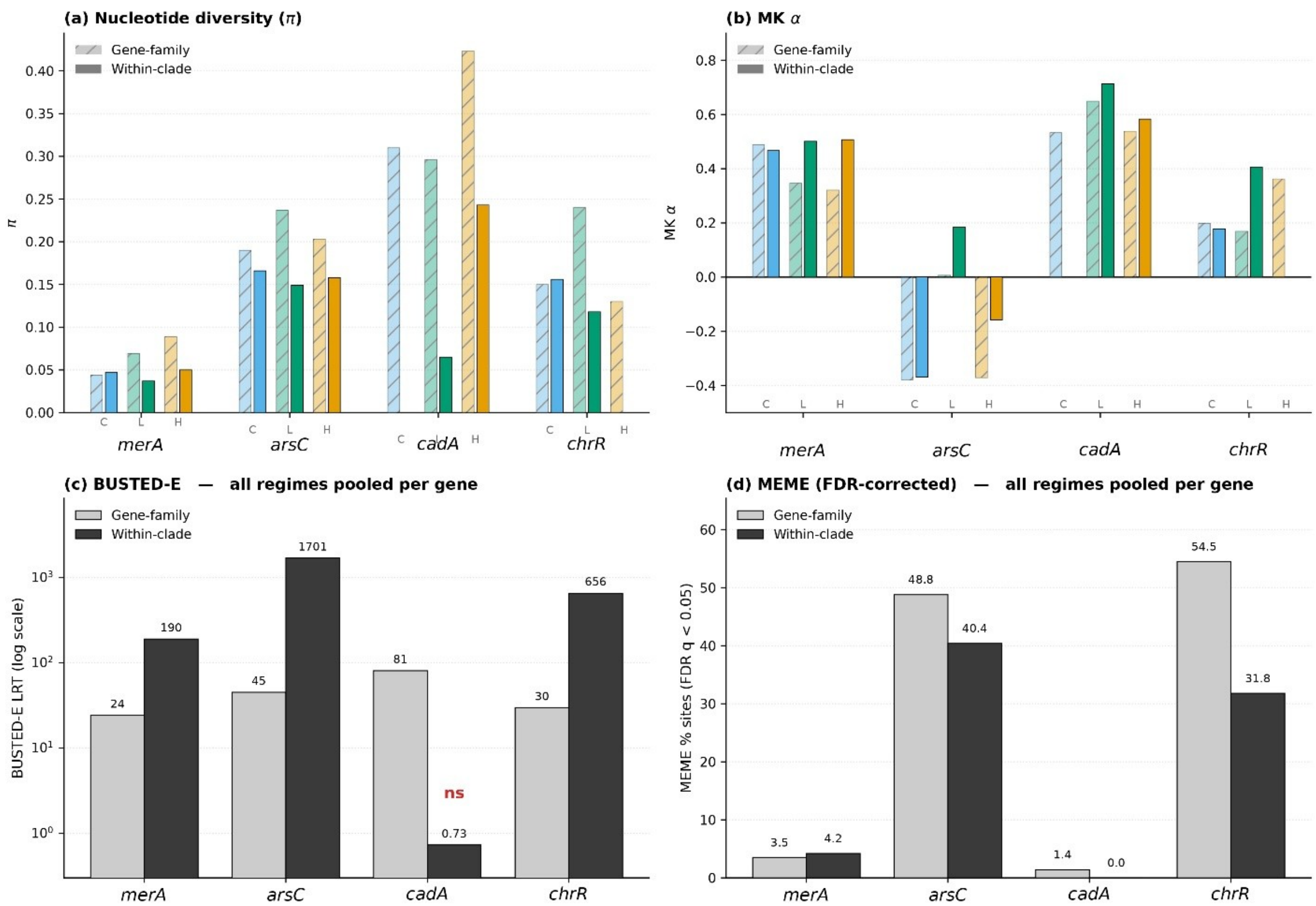

Figure S6. Gene-family vs within-clade comparison across four metrics: (a) π by gene and regime; (b) McDonald-Kreitman α by gene and regime; (c) BUSTED-E LRT (log scale) by gene; (d) MEME % sites under positive selection (FDR $q < 0.05$) by gene. Hatched bars: gene-family analysis (all haplotypes); solid bars: within-clade analysis (largest MMseqs2 ortholog cluster only, Table S12). For panels (a) and (b), micro-labels C/L/H below each pair of bars identify the regime within each gene. For panels (c) and (d), values pool all regimes per gene. The ns annotation in panel (c) indicates non-significance of the within-clade BUSTED-E test for *cadA* (LRT = 0.73, $p = 0.347$).

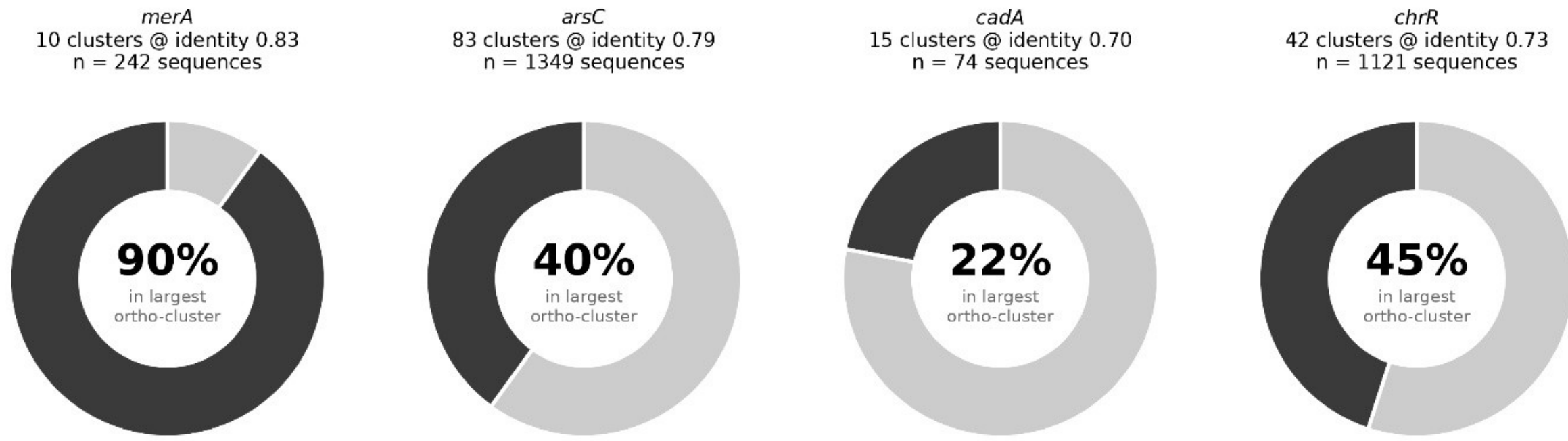

Figure S7. MMseqs2 ortholog cluster summary per gene, displayed as donut charts. The percentage of haplotypes in the largest cluster at the data-driven elbow threshold is indicated in the centre of each donut; the total number of clusters and the elbow threshold are indicated above each gene panel. Numerical values are reported in Table S12.

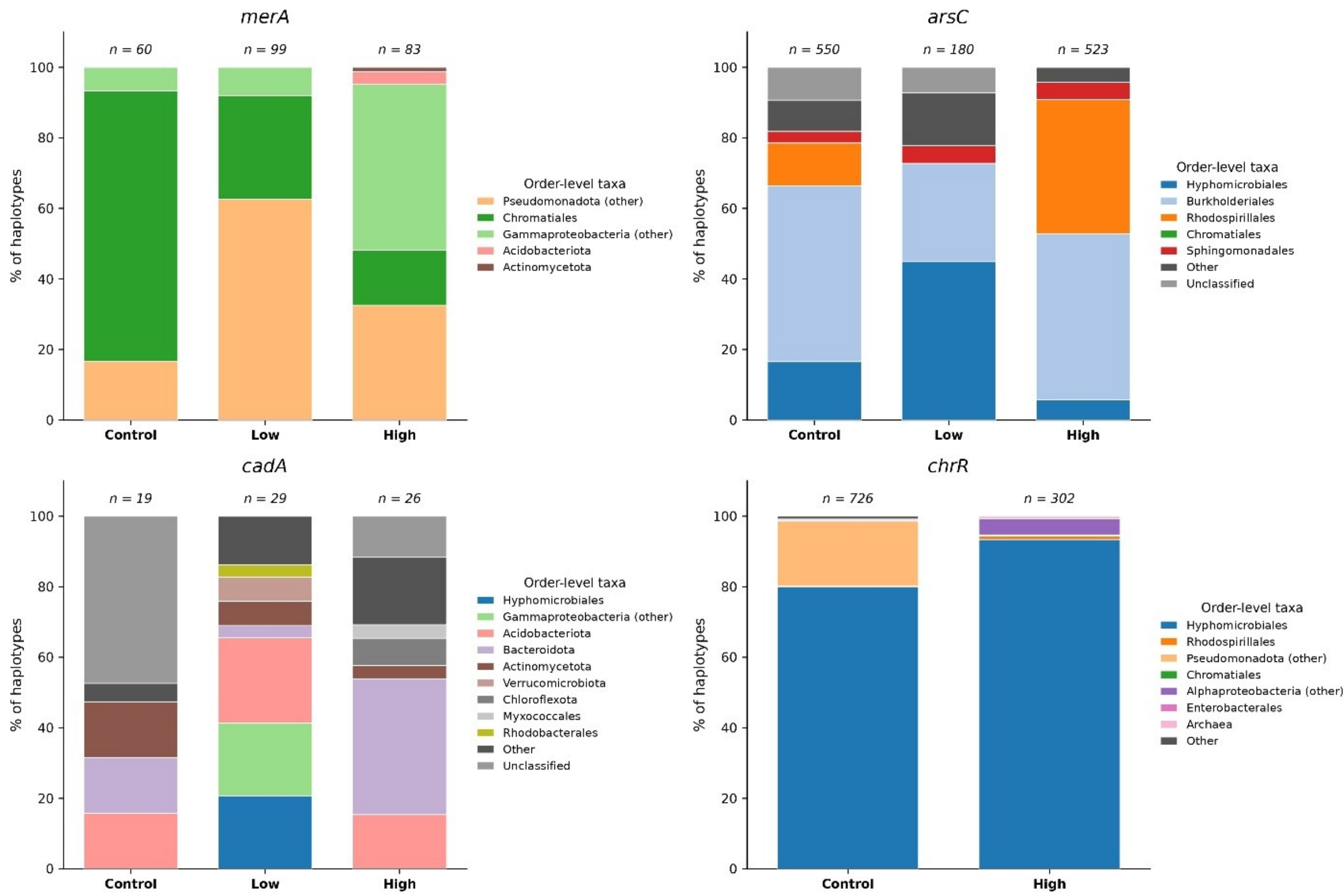

Figure S8. Taxonomic composition of MRG-containing haplotypes per gene and regime, aggregated to the order level for cross-gene comparability. Stacked bars indicate the percentage of haplotypes affiliated with each order, based on best-hit BLASTP against NCBI nr. The total number of haplotypes per regime is indicated above each bar. The *chrR* Low regime is not shown because the 76 Low-regime haplotypes could not be taxonomically assigned within the wall-clock allowance (92% overall coverage; see Methods Section 2.7). Numerical values are reported in Table S13.
